# The effect of intradialytic resistance exercise on physical function and dialysis adequacy in patients on maintenance hemodialysis

**DOI:** 10.1371/journal.pone.0337910

**Published:** 2026-03-13

**Authors:** Raja Boukadida, Mariem Saadaoui, Nesrine Thabet, Mahfoudh Olfa, Fradi Asma, Salsabil Nouir, Wissal Sahtout, Narjes Ben Aicha, Awatef Azzabi, Yosra Guedri, Imed Laatiri, Sanda Mrabet, Dorsaf Zellama

**Affiliations:** 1 Department of Nephrology, Faculty of Medicine of Sousse, Sahloul University Hospital, University of Sousse, Sousse, Tunisia; 2 Department of Paediatrics, Faculty of Medicine of Sousse, Sahloul University Hospital, University of Sousse, Sousse, Tunisia; 3 Department of Physiology and Functional Explorations, Faculty of Medicine of Sousse, Farhat Hached University Hospital, University of Sousse, Sousse, Tunisia; University of Verona: Universita degli Studi di Verona, ITALY

## Abstract

**Background:**

Chronic kidney disease poses a growing global health concern and is linked to several complications with higher prevalence and intensity in the hemodialysis (HD) population. These complications contribute to high morbidity and mortality and are associated with poor physical function, and poor quality of life. Intradialytic exercise has emerged as a promising strategy to improve HD patients’ clinically relevant outcomes.

**Aims:**

Assess the effect of intradialytic exercise on the functional and metabolic status of patients undergoing HD, and on their physical performance and evaluate its safety and feasibility.

**Methods:**

This was a pre-experimental clinical trial conducted between February and August 2024, including adult patients on maintenance HD at Sahloul University Hospital. Patients underwent a supervised intradialytic resistance training twice or three times a week, over a period of 12 weeks.. Dialysis adequacy parameters, physical function, cardiovascular parameters, as well as patients’ nutritional status were assessed before and after the intervention.

**Results:**

Our study included 21 patient with a female predominance (76.2%). The population’s mean age was 44.5 ± 10.4 years. A total of five patients (23.8%) received hemodialysis twice a week, while the remaining 16 patients underwent dialysis three times a week. Over the three-month intervention, the six minutes walk test distance improved significantly with a mean paired difference of 26.4 m (p = 0.007). As for cardiovascular parameters we noted that intradialytic blood pressure decreased from 121.7 mmHg to 112 mmHg (p = 0.03). Dialysis adequacy markers also showed significant increases in creatinine reduction ratio (p = 0.04) and Urea Reduction Ratio (p = 0.04). Furthermore nutritional status showed fewer patients at risk of malnutrition and BMI shifted toward healthier ranges.

**Conclusion:**

Our study results suggest that three months of intradialytic resistance exercise safely improved HD patients’ care including cardiovascular state, physical function and adequacy parameters. Further research especially combining resistance and aerobic exercise is needed to expand and generalize these results.

**Trial registration:**

The trial was retrospectively registered with the Pan African Clinical Trial Registry (PACTR202506776186443).

## Introduction

Chronic kidney disease (CKD) is a progressive condition that affects more than 10% of the general population worldwide, amounting to more than 800 million individuals [1]. End-stage renal disease (ESRD), the last stage of CKD, is an emerging global public health problem and it requires patients to undergo regular renal replacement therapy (RRT). Dialysis is the predominant RRT in most countries, with maintenance hemodialysis (HD) being the most common modality. In 2008 the prevalence of patients undergoing HD was 122 per million population worldwide and 750 per million population in Tunisia and its incidence is growing by approximately 8% annually [2,3]. Progressive CKD is linked to several complications with higher prevalence and intensity in HD population, which interact with each other [4] such as hypertension, cardiovascular complications, anemia, CKD-related mineral bone disorder, salt and water retention, metabolic acidosis and electrolyte disorders, as well as uremic syndrome and poor nutritional status along with skeletal muscle loss [5]. These complications contribute to high morbidity and mortality and poor quality of life and were identified as being relevant to the global burden of poor health caused by CKD [6]. Other complications are well established but difficult to assess thus understudied especially the loss of physical function, associated or exacerbated by the high prevalence of coexisting comorbidities [7]. Despite the current substantial medical and pharmacological advances, and giving the complications above, HD patients continue to have strikingly higher mortality rates compared to age, gender, and race-matched populations and the most important predictors of poor outcome are increasing age, cardiovascular disease, diabetes, and poor nutrition [8]. Higher mortality risk has been reported for sedentary patients [6]. In recent years, the potential of physical exercise as a therapeutic tool in patients with ESRD undergoing maintenance HD treatment has been investigated and research suggested that Increasing activity levels is a promising solution to combat muscle wastage [6,8,9], as it increases dialysis adequacy and other clinically relevant outcomes such as patients’ functional capacity and quality of life [10–13]. Intradialytic exercise [14], defined as any type of exercise performed during dialysis sessions has been proposed as a viable strategy to increase physical activity in this population. As previous studies [11,13] have suggested that intradialytic exercise is effective in enhancing exercise tolerance, improving quality of life and psychological status. Research also indicates that intradialytic exercise can increase the efficacy of dialysis [14,15], subsequently alleviating inflammation, improvingbone mineral density and nutrition.

To date, no study in Tunisia has investigated the impact of intradialytic exercise in patients undergoing maintenance hemodialysis. Previous local research has primarily focused on interdialytic exercise interventions. Given the demographic and cultural specificities of the Tunisian population, the effects of intradialytic training remain largely unexplored. Therefore, our project aimed to:

- Assess the effect of intradialytic exercise on the physical performance of patients undergoing HD as well as their dialysis adequacy.
- Evaluate the safety of intradialytic exercise, as well as its effects, in terms of maintenance HD patient’s clinical outcomes.

## Materials and methods

### Study design

Interventional pre-experimental 3-months clinical trial with supervised intervention of intradialytic resistance exercise training.

### Study population

This study was conducted between February and August 2024 including patients undergoing maintenance HD therapy at the Chronic Hemodialysis Unit in Sahloul University Hospital of Sousse.

**Inclusion criteria:**

- Patients aged over 18 years.
- Undergoing Maintenance HD for at least three months at least twice a week.
- Patients who voluntarily gave a written informed consent.

**Non inclusion criteria:**

- Patients undergoing maintenance HD for less than three months
- History of myocardial ischemia in the last 6 months.
- History of Severe chronic Heart failure (New York Heart Association stage > 3).
- Symptomatic cardiovascular disease with functional disability
- Restrictive lung disease or Chronic Obstructive Pulmonary Disease in advanced stages (GOLD stage III or IV), or during acute exacerbation and Respiratory failure requiring oxygen therapy or non-invasive ventilation.
- Musculoskeletal disorders preventing the patients from performing the exercises.
- Persistent uncontrolled hypertension defined as systolic blood pressure (SBP) ≥ 200 mmHg and/or diastolic blood pressure (DBP) ≥ 120 mmHg.

**Exclusion criteria:**

- Non-adherence to the prescribed four-hour dialysis sessions.
- Acute Condition, throughout the trial period, preventing patients from continuing study protocol (Active infection or sepsis, Recent fractures or trauma …)
- Withdrawal of consent.

### Sample size

All patients meeting the eligibility criteria and who consented were enrolled in the study. A pragmatic recruitment approach was adopted based on the feasibility within the available population; for this reason, no formal sample size was calculated.

### Study protocol

#### General measures.

- The recruitment period extended from February 5 to April 5 2024.
- We initially informed eligible patients in detail about the study objectives, procedures, potential risks, and expected benefits. Written informed consent was then obtained from each participant prior to inclusion.
- The intervention involved exercises conducted a minimum of twice per week over a period of at least 12 weeks, starting in May and concluding on August 5 2024.
- The prescribed exercise duration was one hour, scheduled during the second hour of the 4-hour dialysis session.
- During the dialysis sessions in which the exercise was performed, patients were supervised by a physician and/or a physiotherapist. Blood pressure and heart rate were monitored at least four times—once every hour: immediately before, at regular intervals during, and immediately after the exercise sessions.
- The exercise session was canceled if systolic BP > 200 mmHg, diastolic BP > 110 mmHg or HR > 120 beats per minute.

### Resistance exercise protocol: The intervention

A typical program consisted of eight lower and upper body (Free Hand with no vascular access) resistance exercises using thick-coloured elastic bands (Thera-band) commonly used in physical therapy resistance programs.

We disposed of two different resistance Thera-bands: Medium intensity (3–4 kg) and High intensity (4–6 kg).

Patients initially used medium-intensity bands (3–4 kg resistance). Progression was achieved by increasing the resistance level (from medium to high, 4–6 kg) when patients could comfortably complete all the repetitions sets. The exercises were individually adapted to each patient’s tolerance, and intensity was reassessed every week to ensure gradual and safe progression over the three-month period.

- Upper Limb:

Three sets of 10 repetitions of medium intensity **Elbow extension** then adapted to patient’s tolerance

Three sets of 10 repetitions of medium intensity **Elbow flexion** then adapted to patient’s tolerance

Three sets of 10 repetitions of medium intensity **Shoulder abduction** then adapted to patient’s tolerance

Three sets of 10 repetitions of medium intensity **Shoulder flexion** then adapted to patient’s tolerance

- Lower Limbs:

Three sets of 10 repetitions of medium intensity **Hip flexion** then adapted to patient’s tolerance

Three sets of 10 repetitions of medium intensity **Knee extension** then adapted to patient’s tolerance

Three sets of 10 repetitions of medium intensity **Hip abduction** then adapted to patient’s tolerance

Three sets of 10 repetitions of medium intensity **Hip adduction** then adapted to patient’s tolerance

### Data collection

Demographic characteristics and Patients medical history were collected at baseline. Blood samples for laboratory data were obtained from arteriovenous shunt just before starting the first hemodialysis session of the week at baseline **(T1)** and the immediate week after completing the intervention **(T2).**

Intradialytic systolic blood pressure (SBP), diastolic blood pressure (DBP) and heart rate (HR) were measured four times—once every hour—during each dialysis session involving exercise. Intradialytic weight gain is recorded every session at entry.

To calculate the monthly mean intradialytic SBP, DBP, HR and intradialytic weight gain for each patient, all recorded values from the sessions conducted in a given month were averaged.

It is important to note that the antihypertensive therapy of all participants remained unchanged throughout the study period (which contributed to minimizing potential confounding factors related to blood pressure control).

Patients undergoing hemodialysis twice a week completed approximately 8 sessions per month, while those on a thrice-weekly regimen completed about 12 sessions monthly. Since 4 measures were obtained per session, the total number of values considered for monthly averages was 32 for the twice-weekly group and 48 for the thrice-weekly group. The monthly mean for each parameter was then calculated by summing all respective readings and dividing by the total number of measurements.

Dry weight was determined by considering cardiothoracic ratio, blood pressure, clinical symptoms, and physical findings such as oedema.

Physical Function was evaluated using the 6 minutes walking test (6MWT) at **T1** and **T2**. The test was performed in the same location and in the morning to minimize variability.

Nutritional status, using the Mini Nutritional Scale (MNA), as well as the body composition data and weight were recorded using a bioelectrical impedance analysis (BIA) device (Beurer BF 600, Beurer GmbH, Germany) immediately at the end of the dialysis session at **T1** and **T2**.

The device operates at a measurement frequency of 50 kHz and provides estimates of body fat, muscle mass, and body water. According to the manufacturer, the scale has a maximum capacity of 180 kg, with a weight resolution of 100 g and a measurement precision of 0.1% for body composition parameters.

### Outcomes

- Primary outcome: Improvement of the 6MWT distance from baseline (T1) to post-intervention (T2), assessing functional capacity changes.
- Secondary outcomes:
  - Cardiovascular tolerance assessed by BP and HR trends during dialysis sessions from T1 to T2
  - Improvement of dialysis adequacy parameters (URR and KT/V) from T1 to T2
  - Nutritional status improvement (MNA score, BMI, serum protein levels) from T1 to T2

### Variables definitions

- Six minute Walk test (6MWT)

Patients were asked to walk back and forth a premeasured, outdoor circuit (35m), attempting to cover as much distance as they could in 6 minutes, with the measured distance in meters as the outcome. Heart rate, blood pressure, and oxygen saturation were determined at rest and 6 minutes (peak exercise) [15].

- Functional symptoms such as dyspnea was assessed using the The Visual Analogue Scale **(VAS)**: It consists of a continuous line, 10 cm in length, where **0** represents “no dyspnea” and **10** represents “suffocating”. Patients are asked to mark a point on the line that corresponds to the intensity of their dyspnea at baseline and at 6 minutes [16].
- **Δ6MWT** refers to the change in distance walked during the 6MWT before and after an intervention: Δ6MWT=6MWTPost −6MWTPre

- Dialysis Adequacy:

Defined by the Kidney Disease Outcomes Quality Initiative KDOQI 2015 [17] as the effective removal of waste products, toxins, and excess fluid from the blood during dialysis to maintain optimal patient health and improve clinical outcomes. It is measured primarily using the following parameters:

- **Kt/V**: (**K** = dialyzer clearance of urea (mL/min), **t** = dialysis session duration (minutes) **V** = urea distribution volume) it is recommended a target single pool Kt/V of 1.4 per hemodialysis session for patient treated thrice weekly, with a minimum delivered Kt/V of 1.2.
- **Urea Reduction Ratio (URR)**: Percentage reduction in blood urea during dialysis: It is recommended that the URR consistently >65% and it is calculated as follows:

URR = (Urea pre session −Urea post session)/ Urea pre session ×100

** **Urea pre session** refers to the urea concentration measured immediately before the dialysis session while **Urea post session** refers to the urea concentration measured immediately after the same dialysis session.

- **Creatinine Reduction Ratio (CRR):** Percentage reduction in serum creatinine during dialysis

CRR = (creatinine pre session −creatinine post session)/creatinine pre session ×100

** **Creatinine pre session** refers to the creatinine concentration measured immediately before the dialysis session and **creatinine post session** refers to the creatinine concentration measured immediately after the same dialysis session.

- **Volume Control**: Achieving and maintaining adequate **dry weight** [17,18] defined as the body weight at the end of dialysis at which the patient can remain normotensive until the next dialysis despite the retention of saline and ideally without the use of antihypertensive medications.
- Dialysis adequacy include also Symptom management (e.g., fatigue, pruritus), the assessment of the nutritional status the quality of life and the cardiovascular health and mortality risk reduction [17].
- The mini nutritional assessment (MNA):

It consists of a screening part and an assessment part. The screening part contains 6 items concerning decline of food intake, weight loss in the past 3 months, acute mobility, disease/distress, neuropsychological problems, and additional anthropometric measures [19]. The score indicates three different levels of nutritional status: well-nourished (30–24 points), at risk of malnutrition (23.5–17 points), and malnourished (<17 points). The MNA test was administered at baseline and at the end of the exercise program.

- The body mass index (BMI)

BMI = kg/m2 is the metric currently in use for defining anthropometric height/weight characteristics in adults and for classifying them into groups according to the World Health Organisation standards [20]:

- Underweight <18.5,
- Normal Range 18.50–24.99,
- Overweight ≥ 25
- Obese ≥ 30
  - Adherence Rate is defined as the total number of exercise sessions attempted divided by the total number of exercise sessions offered, multiplied by 100% [21].
  - Bioelectrical impedance analysis or Impedance metry is non-invasive method used to assess body composition, including: Fat mass, Lean body mass (muscle), Total body water (TBW), Bone mass [22]. We used in our study the Beurer BF 600 Bioelectrical Impedance and the normal range for general population is defined as 50–65% of body weight in men and 45–60% of body weight in women for TW, less than 25 and 30% in women and men respectively for Fat mass, and 35–50% of body weight in men and 30–45% of body weight in women for muscle mass.

### Statistical considerations and data analysis

All statistical analyses were performed using the IBM SPSS version 27.0.

Categorical variables were summarized with percentages.

To test the normality of our quantitative variables, we applied the Shapiro-Wilk Test for normality. Quantitative variables following a normal distribution were expressed as mean, standard deviation, and minimum and maximum values. The paired Student’s t-test was used to compare means. Quantitative variables with a non-normal distribution were expressed as median and interquartile range. The Wilcoxon Signed-Rank Test was used to compare medians.

We used Spearman’s test and Pearson’s test to study the correlation between variables. The correlation coefficient 0.00 to ±0.30: was considered a Weak correlation, ± 0.30 to ±0.70: Moderate correlation and ±0.70 to ±1.00 was considered Strong correlation.

In all tests, p values of less than 0.05 were considered statistically significant.

### Ethical considerations

The study’s objectives, as well as participants’ rights regarding participation and withdrawal, were clearly explained to all individuals, and participation was entirely voluntary. Ethical approval was obtained from the Ethics Committee of the University of Medicine of Sousse (Reference: CEFMS 184/2023), in accordance with international standards for research involving human subjects. The study was retrospectively registered at the Pan African Clinical Trial Registry (PACTR202506776186443) due to a lack of awareness at the time that prospective registration was mandatory. Importantly, the protocol had been finalized prior to enrollment, and no changes were made to the design, outcomes, or analyses after registration. The authors confirm that all ongoing and related trials for this intervention are registered.

## Results

### Participants flow

Among our 90 hemodialysis patients, only 60 were receiving maintenance HD at least twice a week. Of these, 30 patients either declined to participate or met non-inclusion criteria. Refusals were often related to personal or logistical reasons, or the belief that therapeutic interventions such as exercise were unnecessary.

**Fig 1:**
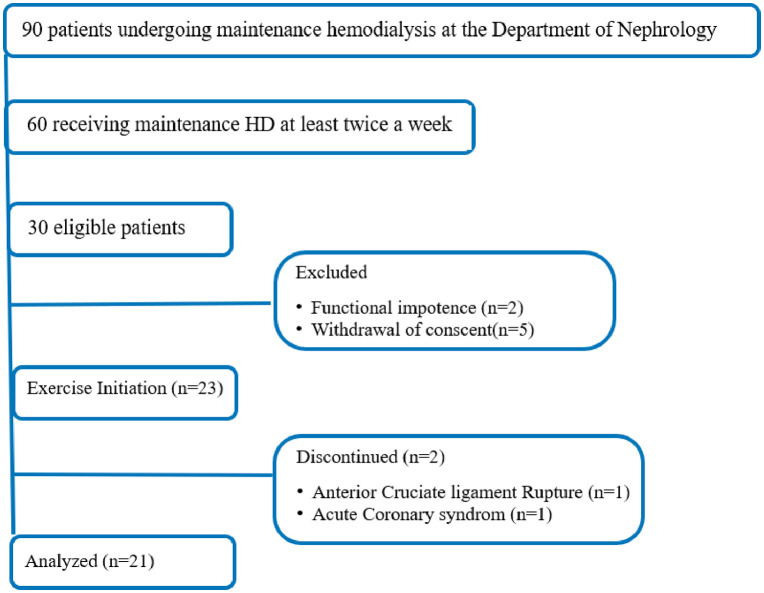
Participants’ Flow Chart.

Thirty eligible patients were approached to participate in the intradialytic resistance exercise program. Seven were excluded due to functional impairment or withdrawal of consent. Twenty-three patients initiated the program, but two discontinued it, resulting in a final sample of 21 patients undergoing maintenance HD at the Chronic Hemodialysis Unit of Sahloul University Hospital, Sousse, between February and August 2024 (Fig 1)

### Sociodemographic characteristics

The mean age for our patients was 44.5 ± 10.4 years with extremes ranging from 27 to 64 years. The distribution of patients according to age groups showed that 44.8% were between the ages of 40 and 50 years.

Our series consisted of 16 women (76.2%) and 5 men (23.8%), resulting in a female-to-male sex ratio of 0.31. Ten patients (47.6%) reported that they were not working.

The Table 1 summarizes patients’ sociodemographic characteristics at baseline.

**Table 1:** Sociodemographic characteristics at baseline.

Variables	Results
**Age — yr (Mean±SD)**	44.5 ± 10.4
**<40 — n (%)**	6 (28.6)
**40-50 — n (%)**	9 (44.8)
**>50 — n (%)**	6 (28.6)
**Female sex — n (%)**	16 (76.2)
**Smoking Status — n(%)**	
**Former**	1 (4.8)
**Current**	1 (4.8)
**Never**	19 (90.4)

n:number, SD: standard deviation, yr: years.

### Clinical characteristics

In our study population, Hypertension was the most common comorbidity, affecting 52.4% of patients, with an average duration of 9.1 ± 5.6 years. Diabetes was present in 14.3% of cases. Parathyroidectomy, was performed in 19% of patients. While coronary heart disease was present in one patient (4.8%) and three patients had chronic hepatitis. The most represented nephropathy was chronic interstitial nephritis in 52.4% of cases. Glomerular nephropathy was present in 38.1% of cases, in which diabetic nephropathy was present in 14.3% of patients. Residual urine output was present in 38.1% of patients. Two patients had a history of renal transplantation.

These patient’s comorbidities are resumed in Table 2.

**Table 2:** Comorbodities Distribution.

	Effective (%)	Duration —yr (Mean±SD)
**Hypertension**	11 (52.4)	9.1 ± 5.6
Parathyroidectomy	4 (19)	–
**Diabetes**	3 (14.3)	33 ± 3.6
**Chronic Hepatitis**	3 (14.3)	–
**Coronary Heart Disease**	1 (4.8)	–
**Etiology of the Chronic Kideny Disease**		
**Interstitial**	11 (52.4)	
**Glomerular**	8 (38.1)	
**Diabetic**	3 (14.3)	
**Vascular**	1 (4.8)	
Indeterminate	1 (4.8)	
Renal transplantation	2 (9.5)	

n:number, SD: standard deviation, yr: years.

### Dialysis parameters

All patients in our study adhered to the four-hour dialysis session duration. A total of five patients (23.8%) received hemodialysis twice a week, while the remaining 16 patients underwent dialysis three times per week. These parameters are summarized in the Table 3.

**Table 3:** Characteristics of Dialysis Access, Vintage, and Treatment Parameters.

Characteristics	Values
**Dialysis Vintage — yr (Median [IQR])**	6 [5–8.5] (min: 1-max: 20)
**Type of vascular access (n, %)**	
**Permanent**	20 (95.2)
**Arteriovenous fistula**	19 (95)
**Gore-Tex**	1 (4.8)
**Temporary: Jugular vein catheter**	1 (4.8)
**Blood Pump Rate (mL/min), (Mean±SD)**	280 ± 18.9(min: 240-max: 300)
**Dialyzer surface (m²), (Mean±SD)**	1.7 ± 0.9 (min: 1.4-max: 1.7)

n:number, SD: standard deviation, yr: years, IQR: interquartile range, min: minimum, max:maximum.

### Outcomes

#### Primary outcome.

**Fig 2:**
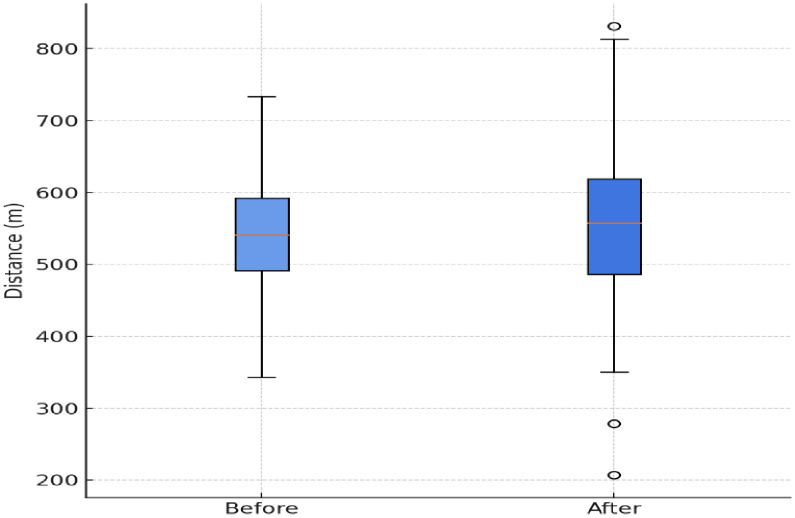
The 6MWT distance distribution before and after trial.

The 6-minute walk test (6MWT) distance walked by our study participants showed a statistically significant increase from 516.4 ± 103.4 meters to 563.3 ± 104.3 meters **(p = 0,007)**, with a mean paired difference of 26.4 meters after the 3-month intervention period. The Fig 2 displays the changes in the 6MWT distance over trial period. The mean values of the performance achieved during the 6MWT are presented in  Table 4.

**Table 4:** The 6 MWT Tolerance parameters.

		T1	T2	p
6 MWT distance—m (Mean±SD)		516.4 ± 103.4	563.3 ± 104.3	0.007
SpO2—% (Mean±SD)	**Before 6MWT**	97.8 ± 1.5	97.7 ± 1.5	0.69
	**After 6MWT**	96.8 ± 1.7	96.5 ± 1.7	0.57
SBP —mmHg (Mean±SD)	**Before 6MWT**	124.8 ± 22.9	120.5 ± 22.5	0.61
	**After 6MWT**	150 ± 35.2	134.7 ± 22.5	0.12
DBP —mmHg (Mean±SD)	**Before 6MWT**	75.2 ± 12.5	70.5 ± 11.8	0.48
	**After 6MWT**	83.8 ± 17.5	80 ± 11.5	0.07
Heart Rate —bpm (Mean±SD)	**Before 6MWT**	85.3 ± 16.5	83.7 ± 9	0.28
	**After 6MWT**	102.6 ± 22.9	98.5 ± 14.9	0.19
VAS (Median[IQR])	**After 6MWT**	3 [2 –5]	5 [3 –5]	0.97

SD: standard deviation, IQR: interquartile range, 6 MWT: 6-minute walk test, m: meters, SBP: systolic blood pressure, DBP: Diastolic blood pressure, VAS: Visual Analogue Scale.

There were no statistically significant changes in the 6MWT tolerance parameters. Specifically, systolic blood pressure (SBP)and diastolic blood pressure (DBP) showed no significant changes either before or after the 6MWT (SBP: p = 0.61 and 0.12; DBP: p = 0.48 and 0.07). Similarly, perceived dyspnea as measured by The Visual Analogue Scale (VAS) remained almost unchanged (p = 0.97). (Table 4)

**Fig 3:**
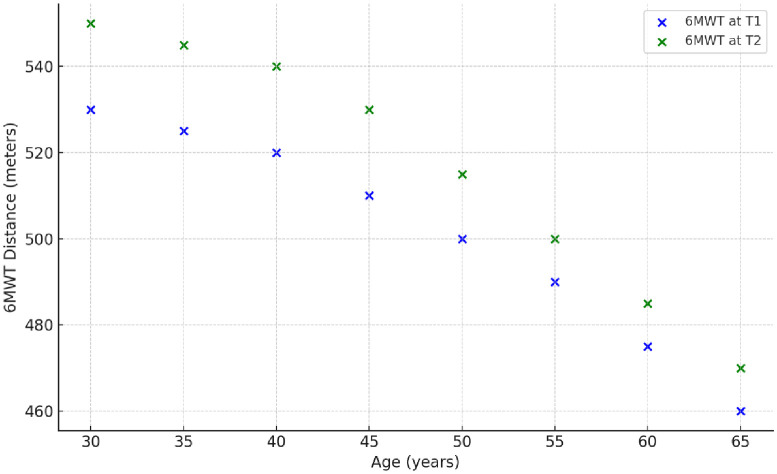
Scatter plot of 6MWT distance vs Age.

As displayed in the Fig 3, at T2, age was moderately to strongly negatively correlated with 6MWT.

### Secondary outcomes

#### Cardio-vascular parameters.

The Table 5 displays the means of averaged monthly cardiovascular parameters recorded during sessions in the first (T1) and third month (T2) of our trial period.

**Table 5:** Monthly Trends in Dialysis cardiovascular Parameters.

	T1	T2	p
Intradialytic SBP mmHg (Mean±SD)	121.7 ± 22.6	112 ± 28.9	**0.03**
Intradialytic DBP mmHg (Mean±SD)	71.9 ± 12.6	67.6 ± 10.9	0.07
Interdialytic Weight gain, Kg, (Mean±SD)	2.8 ± 0.7	2.8 ± 0.8	0.62
Heart rate bpm (Mean±SD)	85.9 ± 17.5	85 ± 9.4	0.82

SD: standard deviation, IQR: interquartile range, 6 MWT: 6-minute walk test, m: meters, SBP: systolic blood pressure, DBP: Diastolic blood pressure.

Mean intradialytic Systolic blood pressure dropped gradually from the first month with a mean of 121.7 mmHg to 112 mmHg in the third month of intervention. Also mean intradialytic diastolic blood pressure dropped from a mean of 71.9 mmHg to 67.6 mmHg.

### Hemodialysis adequacy

Hemodialysis dose: Dialysis dose parameters variations between T1 and T2 are displayed in the Table 6, we noted that urea levels significantly decreased both pre-and post-dialysis (p < 0.001).

**Table 6:** Hemodialysis solute clearance parameters at T1 and T2.

	T1	T2	p
Urea pre session—mmol/l (Mean±SD)	32.3 ± 6.7	27 ± 7.7	**<0.001**
Urea post session—mmol/l (Mean±SD)	9.8 ± 3.4	7.2 ± 2.8	**<0.001**
Creatinine pre session—µmol/l (Mean±SD)	1152.1 ± 224.9	1192.8 ± 280.5	**<0.001**
Creatinine post session—µmol/l (Mean±SD)	403.8 ± 133.5	370.3 ± 114.7	**<0.001**
Creatinine reduction ratio—% (Mean±SD)	64.9 ± 7.1	68.5 ± 5.4	**0.04**
URR —% (Mean±SD)	69.4 ± 8	73.6 ± 5.2	**0.04**
KT/V (Mean±SD)	1.5 ± 0.3	1.6 ± 0.2	0.13
Beta-2 microglobulin (Median [IQR])	45[35–70.7]	24.2 [21.4–28.5]	**<0.001**
Potassium pre session—mmol/l (Mean±SD)	5.9 ± 1	5.4 ± 0.9	0.18
Potassium post session—mmol/l (Mean±SD)	3.7 ± 0.3	3.3 ± 0.3	**<0.001**

SD: standard deviation, IQR: interquartile range, URR: Urea reduction ratio.

Also, post-dialysis levels of beta-2 microglobulin and creatinine decreased significantly (p < 0.001).

Results also showed increases dialysis dose parameters; creatinine reduction ratio (p = 0.04) and urea reduction ratio (URR) (p = 0.04).

**Minerals and bone disorders:** This Table 7 illustrates the changes in minerals and bone biochemical indicators before and after the intervention. Phosphorus levels showed a statistically insignificant decrease (2.2 ± 0.5 to 2 ± 0.5, p = 0.22). Calcium levels significantly increased from 2 ± 0.3 to 2.2 ± 0.2 (p < 0.001). Alkaline phosphatase (PAL) levels remained unchanged.

**Table 7:** Changes in Phosphocalcic parameters.

	T1	T2	p
**Phosphorus—mmol/l (Mean±SD)**	2.2 ± 0.5	2 ± 0.5	0.22
**Calcium—mmol/l (Mean±SD)**	2 ± 0.3	2.2 ± 0.2	**<0.001**
**PTH—pg/mL (Median [IQR])**	644[405-983.7]	720 [499.5–1085.3]	0.20
**VITD—ng/mL (Median [IQR])**	18.8[11.3–24.6]	40.5 [26.4–76.5]	**<0.001**
**PAL —U/L (Median [IQR])**	156 [109–257]	157 [107.5–239.5]	0.75

SD: standard deviation, IQR: interquartile range, PTH: Parathyroid Hormone, VitD: vitamin D, PAL: alkaline phosphatases.

### Nutrition and body composition

BMI categories: The Table 8 shows the BMI categories’ shifts throughout the trial period. After the intervention period we noted an increase in the healthy range from 57.1% to 61.9% and a decrease in the overweight category from 33.3% to 23.8% but one patient shifted to the obesity category.

**Table 8:** Population distribution by BMI categories.

BMI Category	T1	T2
**Underweight (n, %)**	2 (9.5)	2 (9.5)
**Healthy range (n, %)**	12 (57.1)	13 (61.9)
**Overweight (n, %)**	7 (33.3)	5 (23.8)
**Obesity (n, %)**	N/A	1 (4.8)
**Total (N, %)**	21 (100)	21 (100)

BMI: body mass index, n: number, N/A: not applicable.

Body composition measurements: Total body water percentage significantly increased from 51.7% to 56.2% (p < 0.001), and fat mass decreased from 25.9% to 23.1% (p = 0.03). These Changes are illustrated in the Table 9.

**Table 9:** Anthropometric measurements before and after trial period.

Parameters	T1	T2	P
Dry weight —Kg (Mean±SD)	61.4 ± 11.4	61.2 ± 11.8	0.72
Weight —Kg (Mean±SD)	63.6 ± 12.1	61.7 ± 11.7	0.10
BMI—kg/m² (Mean±SD)	23.3 ± 3.9	22.8 ± 3.7	0.11
TBW —(%)	51.7 ± 7.3	56.2 ± 6.3	<0.001
Fat Mass —Kg (Mean±SD)	25.9 ± 10.2	23.1 ± 8.8	0.03
Muscle Mass— Kg (Mean±SD)	34.9 ± 7.4	33.5 ± 7.9	0.14

BMI: body mass index, n: number, TBW: Total Body Water, SD: Standard Deviation.

Mini nutritional assessment MNA: Nutritional status exhibited an improvement with the percentage of participants classified as having normal nutritional status increasing from 33.3% to 52.4%, and those at risk of malnutrition decreasing from 61.9% to 47.6%, while the malnourished category disappeared as displayed in the Table 10.

**Table 10:** Population distribution by MNA Nutritional Status.

	T1	T2
**Normal Nutritional Status (n, %)**	7 (33.3)	11 (52.4)
**At risk of malnutrition (n, %)**	13 (61.9)	10 (47.6)
**Malnourished (n, %)**	1 (4.8)	N/A

N/A: not applicable, n: number, MNA: Mini Nutritional Assessment.

Nutritional laboratory markers: The Table 11 summarises the biological nutritional parameters of our population before and after the trial period. Total protein increased significantly from 69 ± 4.6 g/dL to 71.3 ± 6.1 g/dL (p = 0.02) and the rest of the changes were statistically insignificant.

**Table 11:** Population’s biological nutritional parameters changes.

	T1	T2	p
**Albumin —g/dL (Mean±SD)**	38.7 ± 2.8	38.1 ± 2.4	0.21
**Total protein —g/dL (Mean±SD)**	69 ± 4.6	71.3 ± 6.1	**0.02**
**Prealbumin—mg/L (Mean±SD)**	334.3 ± 50.2	–	
**Total Cholesterol —mmol/L (Mean±SD)**	4.2 ± 0.9	4.4 ± 0.9	0.74
**Triglycerides—mmol/L (Mean±SD)**	1.4 ± 0.6	1.6 ± 0.8	0.45
**HDL-Cholesterol—mmol/L (Mean±SD)**	1 ± 0.2	1.3 ± 0.8	0.09

N: Number, SD: Standard deviation.

### Adherence

#### Adherence rate.

Adherence rate had a mean of Average 63.8% ± 27.7 and fifteen Patient (71.4%) had an adherence rate of more than 50%.

#### Reasons of non adherence.

The reasons for non-adherence to intradialytic resistance exercise among our study participants include fatigue (42.9%), personal reasons (28.6%), and medical issues (9.5%).

#### Adverse events and mortality.

During the intervention period, two patients experienced adverse events while exercising: intradialytic hypertension and muscle cramps. There were no reported fatalities associated with the exercise.

## Discussion

Our study **sample size** was 21 patients. In fact, we conducted a single-center study, that’s why recruitment was constrained by the available patient pool. Additionally, we were limited by strict inclusion and exclusion criteria, focusing on relatively healthy dialysis patients and excluding frail individuals and those with significant comorbidities. Thus, out of 30 eligible patients, 5 patients withdrew their consent. According to current literature, sample sizes in intradialytic resistance exercise studies vary widely due to differences in patient populations and study designs. In a systematic review published by Verrelli et al., (2024) [23] investigating the effect of intradialytic exercise on cardiovascular outcomes in adults undergoing maintenance hemodialysis, that included 32 studies from 5 continents, the average number of participants per study was 82.6 ± 51.3 (range: 18–214). Meanwhile, the average participant number in the five Single-Group Pre/Post Design studies included in this systematic review was 22.6 ± 4.77 (range: 18–30), which was similar to our study. It should also be noted that within our sample, 6 participants performed two sessions per week while 15 performed three sessions per week. Given the small number of patients in the twice-weekly subgroup, we analysed all participants together; however, this heterogeneity in training frequency may have influenced the results and is acknowledged as a limitation of our study.

**Choice of *Exercise timing and modalities.*** Intradialytic exercise has been studied as an effective use of the intradialytic period and to incorporate physical activity in Hemodialysis patients under professional supervision. Both types of aerobic and resistance exercises have been thoroughly studied and current literature promotes the combination of aerobic and resistance training as more effective than resistance alone or aerobic training alone to improve functional performance [24]. In our Study We chose intradialytic resistance exercise because of its practicality, affordability, and availability with proven similar effectiveness. A duration of 8 weeks with 2–3 exercise sessions a week corresponding with patient’s twice/thrice-weekly dialysis schedule has been the modality chosen by most of the RCT reviewed. The prescribed exercise duration was 60 minutes during the second hour of a 4-hour dialysis session. In fact previous investigations [25] showed that three 15-minute bouts of exercise during HD were insufficient to cause a detectable increase in serum urea removal and that two 30-minute bouts of exercise substantially elevated the amount of urea removed in dialysate fluid.

The **choice of the 6 minutes walk test** to assess physical function in our trial setting is justified by its wide use on this particular group of patients. In fact, a meta-analysis published by Clarkson et al. (2019) [26], evaluating the effect of exercise interventions on objective physical function in patients on dialysis, found that the 6MWT was utilized in 16 out of the 27 (64%) studies included. Also, the 6MWT was found to be valid and reliable in this setting. Additionally, it is a simple, inexpensive and non-invasive test that is well tolerated and representative of the daily physical efforts that haemodialysis patients make. A systematic review by Martins et al. (2021) [27] concluded that poor 6MWT performance is associated with higher morbidity and mortality and that the distance covered in this test is strongly correlated with cardiorespiratory fitness, muscle strength, and overall quality of life [28,29].

### Sociodemographic characteristics

The mean age of our patients was 44.5 ± 10.4 years, with 44.8% of them aged between 40 and 50 years. In the current literature, there is a disparity in the mean age of study populations, which varies according to the exercise modality. Studies evaluating the effect of intradialytic aerobic exercise tend to have a younger populations, followed by studies assessing resistance respiratory exercises [23,25]. In studies similar to our trial setting, age variations have been observed. A quasi-experimental study by Baião et al. (2024) [28] included 18 patients with a mean age of 62 years, with 55.6% aged 60 or older. However, in the study conducted by Marzougui et al. (2022) [29] the mean age was 43.76 ± 12.56 years, which is comparable to our study.

Our series consisted of 16 women (76.2%) and 5 men (23.8%), resulting in a female-to-male sex ratio of 0.31. Reviewing the current literature we noted a male predominance (23,25,29). The closest gender distribution to our study was the study conducted by Martin-Alemañy, et al. (2016) consisting of 34.1% male and 65.9% female [27].

Ten patients (47.6%) reported that they were not working at time of the trial. These findings align with known epidemiological data on CKD patients, especially those on maintenance hemodialysis, which disproportionately affects economically disadvantaged individuals due to limited access to healthcare [30].

The majority of our study population (90%) reported they had never smoked. However based on recent studies Smoking prevalence in maintenance hemodialysis patients is notable, 9.4% are current smokers and 16.7% are former smokers in this multicenter cross-sectional study by Fadili et al. (2023) [31].

### Clinical characteristics

Regarding our participants comorbidities; Hypertension was the most common condition, affecting 52.4% of patients, with an average duration of 9.1 ± 5.6 years. Followed by diabetes in 14.3% of cases, lasting 33 ± 3.6 years. Parathyroidectomy, was performed in 19% of patients. While coronary heart disease was present in one patient (4.8%) and three patients had chronic hepatitis. Our study findings aligned with recent investigations [23], confirming the high prevalence of hypertension among HD patients, which varies across studies, with rates ranging from 23% to 100%. Diabetes was reported with a prevalence ranging from 0% to 43% of participant [32], depending on study inclusion criteria. Coronary artery disease was present in some studies’ populations, with rates between 10% and 47%. Other studies excluded participants with infections diagnosed within the previous six months.

In our trial setting the most predominant nephropathy was chronic interstitial nephritis in 52.4% of cases. Followed by glomerular nephropathy in 38.1% of cases, among which diabetic nephropathy was present in 14.3% of patients. In our population the mean CKD progression was 9.2 ± 4.8 years and patients who still have Residual urine output represented 38.1% of our participants. As evidenced by current research most of the reviewed studies do not specify the etiology of CKD nor the presence of a residual urine output among participants, suggesting that these variables may not be strongly correlated with resistance exercise outcomes.

All patients in our study adhered to the four-hour dialysis session vintage. A total of five patients (23.8%) received hemodialysis twice a week, while the remaining 16 patients underwent dialysis three times per week. Many studies had the same dialysis sessions duration and frequency especially the one conducted by Yabe, et al. (2021) [33] and the study conducted by Ribeiro HS, et al. [34]. In contrary, in the trial conducted by Baião, et al. [28] patients underwent HD sessions five to six times per week, with each dialysis session lasting 2 h or 2 h30 min.

The study population had a mean dialysis duration of 7.1 ± 4.2 years (range: 1–20 years). Studies similar to our study design such as the one conducted in 2021 by Kato M, et al [35].

Dialysis vintage ranged from 1 to 200 months (48.3 ± 52.8 months). The Majority of our study participants (95.2%) were using permanent vascular access, primarily AV fistulas (95%), while 4.8% relied on temporary jugular vein catheterization comparable to the results found by Baião, et al. [28]. The mean blood pump rate was 280 ± 18.9 mL/min (range: 240–300 mL/min), and the mean dialyzer surface area was 1.7 ± 0.9 m², with most patients using 1.7 m² dialyzers.

### Outcomes

Regarding our study physical function results, at baseline our participants covered 516.4 ± 103.4 m on the six minutes walk test (6MWT). These results exceeded literature reviewed results. In Fact, the 6MWT distance covered by hemodialysis patients, widely different across various studies, ranges approximately from 325 to 450 meters [26]. These differences may be attributed to the young mean age of our population distribution, our inclusion and exclusion criteria. Additionally, they may be influenced by the imbalanced gender distribution, with 76.2% of our study population being female.

After intervention protocol participants scored a statistically significant increase in the 6MWT distance from 516.4 ± 103.4 meters to 563.3 ± 104.3 meters **(p = 0,007)**, with a mean paired difference of 26.4 meters. These results align with the findings of Exel, et al., (2021) [36] in a randomized controlled trial of 107 patients who underwent resistance exercise versus stretching program for 8 weeks, three times a week. They found that exercise group significantly improved the 6MWT distance. Comparable to our results, the mean paired difference recorded was 26.27 meters. Another randomized controlled pilot study by Kirkman, et al. (2019) [10] concluded that a 12-week intradialytic resistance training program three times per week, improves 6MWT distance from 532 ± 95 m to 571 ± 101 m Post-intervention with a difference of 41m. Other studies included in a systematic review by Neto et al. (2018) [37] comparing resistance exercise to control group also concluded to statistically significant improvements in the 6MWT distance with a difference of 30.2 m for participants in the exercise training.

Scoping current literature, we noted a key concept which is The minimal clinically important difference (MCID) for the 6MWT. In fact, establishing an MCID helps bridge the gap between statistical significance and clinical relevance, ensuring that interventions lead to tangible improvements in patients’ lives and allowing healthcare providers to tailor exercise programs effectively to enhance patients’ outcome [38]. Although, the 6MWT MCID in hemodialysis patients is not universally established. A systematic review published by Bohannon and Crouch, 2017 [39] identified an MCID range of 14.0 to 30.5 meters across various patient populations, with different pathologies but no specific data were found for CKD patients. Our study paired distance change of 26.4 meters falls within this range, suggesting that resistance exercise led to functionally meaningful benefits for our participants. Similarly, in a large multicenter randomized clinical trial, Manfredini et al. (2017) [40] reported that the 6MWT increased by +39 m in the exercise group versus +2 m in controls in the intention-to-treat analysis, and by +41 m versus +3 m in controls in the per-protocol analysis (P < 0.001 for both). In line with this, Hatef et al. (2017) [41] suggested that a change of approximately 30–50 meters in the 6MWT may be clinically significant for this population. Our study participants showed an overall good tolerance to the 6MWT. Throughout the study period, the oxygen saturation levels remained unchanged and there was no recorded desaturation (p > 0.05). Also, we recorded a mild rise in the systolic blood pressure after exercise, more pronounced at T1 (p = 0.12) with no severe hypertension peaks recorded. Similarly, diastolic blood pressure slightly increased after the 6MWT at T2 but did not reach statistical significance. Heart rate also showed a minimal increase after exercise while staying within safe levels (p = 0.19). However, the VAS perceived dyspnea increased after 6MWT assessment at T2, but did not reach statistical significance (p = 0.969). We hereby suggest that the 6MWT is tolerable among our HD patients, although it can be associated with increased dyspnea which align with literature findings. In fact, Pajek et al. 2016 [42] concluded that ESKD without any other confounding effect of comorbidity is a significant negative predictor of perceived level of dyspnea, irregardless of exercise.

The differences observed in the 6MWT distance compared to our trial results may be attributed to missing data. In fact, three patients declined to undergo the 6 MWT post-intervention which may have impacted our results. Also, patient’s compliance to exercise sessions differed widely across different participants. These results can also be influenced by various factors, including demographic characteristics, comorbidities, Anemia, Nutritional Status, and dialysis dose.

In our study, we noted moderate to strong negative correlation between age and the 6MWT distance (−0.591, p = 0.010) with a weak negative correlation with the 6MWT distance variability Δ6MWT over trial period. Our results are comparable to Franco et al., 2020 [15] who suggested that older patients had significantly lower 6 MWT distance and lower improvement in physical function after exercise and that Males performed longer walking distances on the 6MWT.

Throughout our trial period, intradialytic Systolic blood pressure dropped significantly from the first month with a mean of 121.7 mmHg to 117,2 in the second month and to 112 mmHg in the third month **(p = 0,036)** with no episode of hypotension recorded. We also found that diastolic blood pressure has dropped from a mean of 71.9 mmHg to 67.6 mmHg but it was statistically not significant (p = 0.071). These results were comparable to the current literature. A quasi-experimental study carried by Agustin, et al. (2022) [43] including 30 dialysis patients; 15 patients had underwent resistance exercise twice a week for 30–45 min at the 1st and 2nd hour after the installation of vascular access, for a duration of 8 weeks vs 15 patients in the control group, reported a significant decrease (p = 0.025) in systolic blood pressure from 162.20 mmHg to 153.13 mmHg post-exercise. This study reported however a significant decrease in the diastolic blood pressure from 107 mmHg to 94.33 mmHg post exercise. Additionally, a pre-experimental trial conducted by Hariyanto, et al. (2021) [44] concluded that Intradialytic exercise significantly reduced both systolic (173 ± 18.38 to 160 ± 25.34) and diastolic (99 ± 15.18 to 91.50 ± 10.40) blood pressure, with a p-value of 0.003 and 0.015 respectively. Some studies indicate that the timing and type of exercise may influence blood pressure outcomes which can be a possible explanation of the lack of significance in the diastolic blood pressure reduction in our study. As for interdialytic weight gain and Heart rate no changes were observed during the entire intervention period with respective means of 2.8 ± 0.7 Kg and 85.9 ± 17.5 bpm. Contrary to our study results of some studies concluded that Intradialytic exercise has been effective in reducing interdialytic weight gain. Pujiastuti et al., (2020) [45] reported decreases from 1.438 to 0.281 kg after consistent exercise interventions. Vogiatzaki et al., (2022) [46] concluded to a significant reduction in interdialytic weight gain from 4.79% to 0.15% after implementing a structured intradialytic exercise program.

Moreover, no patient throughout our trial period had experienced chest pain and no patient had exhibited EKG modifications.

To consistently evaluate dialysis adequacy parameters, our study participants adhered to the prescribed four hours dialysis sessions and maintained their usual dialysis settings previously detailed. Blood Pump rate, and Dialyzer surface remained unchanged throughout the trial period.

To evaluate Dialysis dose The KDOQI guidelines [17] currently describe small solute clearance, especially Kt/V, as the best measure of hemodialysis and its adequacy. At baseline, our participants KT/V was at mean of 1,5 ± 0,3 while their URR was at an average of 69,4% ± 8 and the Creatinine reduction ratio was 64.9% ± 7.1. According to current guidelines our patients’ dialysis dose at baseline was overall satisfactory: more than 1.4 for single pool Kt/V per hemodialysis session for patient treated thrice weekly with a minimum delivered Kt/V of 1.2 (17). As for the URR it is recommended to have a minimum of >65%, the case for our study participants, with an optimal range of 70–75%. Calculation of Creatinine reduction ratio is not recommended but it was used by various studies evaluating the effect of exercise on dialysis adequacy, thus, its relevance in our trial setting is clear. After trial period, our patients exhibited statistically significant improvements in small solute clearance: pre-and post-session Urea concentration dropped considerably (p < 0.001). And while Creatinine levels significantly increased pre-session and decreased post-session (p < 0.001), its clearance significantly improved (p < 0.001) after the trial period. Furthermore, we noted a statistically significant reduction in potassium levels post hemodialysis session but no changes were noted in the pre-session potassium levels. As for dialysis dose parameters we noted a statistically significant improvement in the URR, going from 69% to 73% after trial period (p = 0.04) and in the creatinine reduction ratio (p = 0.04). Whereas the Kt/V improved from 1.47 to 1.61, but it was not statistically significant (p = 0.13). Compared to the current literature, our results were consistent with the quasi-experimental conducted by Elshinnawy et al., 2024 [47] involving two groups of patients undergoing regular hemodialysis concluded that intra-dialytic exercise group showed significantly lower blood urea levels and higher URR compared to the control group. Equilibrated Kt/V values were higher in the exercise group as well. Elshinnawy et al. [47], additionally, did not report significant difference in potassium levels between the groups. Brown PDS et al., (2017) [11] also found that intradialytic exercise significantly increased urea clearance compared to no exercise (p < 0.05) and that Kt/V was slightly, but significantly greater compared to no exercise group. These results were further supported by the systematic review of Ferreira GD et al., (2019) [48] concluding to improvement in dialysis adequacy parameters: Kt/V, urea clearance and creatinine reduction ratio in intradialytic exercise patients, while intradialytic intervention did not significantly impact potassium removal.

The differences observed in our study results regarding the insignificant improvement in the Kt/V parameter may be attributed to the loss of a permanent vascular access, AV fistula, in one patient during intervention period replaced with a jugular vein catheter in this one patient impacting our study results.

As For bone and mineral metabolism markers for our population, While phosphorus levels decreased slightly but not significantly (2.2 to 2.0 mmol/L, p = 0.22), calcium levels significantly increased (p < 0.001). These results were also comparable to those of Elshinnawy et al. [47] who found that after a 3-month exercise program, participants exhibited improved calcium and phosphorus balance. We also noted an important statistically significant increase in the participants’ vitamin D levels (p < 0.001), a stability in the Alkaline phosphatase (PAL) levels, while Parathyroid hormone (PTH) levels increased, but this difference did not reach statistical significance (p = 0.205). Scoping current literature, a randomised controlled trial by Tabibi et al, (2023) [49] including 44 participants randomised to 6 months of intradialytic exercise (n = 22) or control (n = 22) concluded to a significant increase in serum calcium (P < 0.05) as well as a significant decrease in serum phosphorus, parathyroid hormone and alkaline phosphatase (P < 0.05). However, another pre-experimental study by Marinho SM, et al. (2016) [50] including 21 patients: 10 patients participated in a resistance exercise training program and 11 patients in the control group, found a reduction in serum phosphorus levels and PTH levels in the exercise group but this reduction was not significant. The increase in PTH levels in our study participants compared to the current literature may have various explanations. For example, none of the above studies specified hemodialysis session prescriptions, for instance, dialyser and/or dialysate calcium concentration may influence calcium, phosphorus balance and PTH secretion. In addition, a recent study by Feng et al. 2025 [51] concluded to changes in the dynamics of bone remodeling in the setting of intradialytic exercise, which may explain a temporary increase in PTH to maintain calcium homeostasis.

Regarding anemia, participants’ hemoglobin (Hb) levels at baseline were 9.1 ± 1.3 g/dl. These levels are insufficient according to current KDOQI guidelines [17] which define anemia as Hb < 11 g/dL in adult dialysis patients with targets between 11–12 g/dL, avoiding levels >13 g/dL.

After intervention period, hemoglobin levels and hematocrit levels slightly but not significantly increased (*p* = 0.34, *p* = 0.57). While the erythropoietin (EPO) requirement remained unchanged. Ferritin levels significantly increased (*p* = 0.03) with an unchanged Intravenous iron administration (*p* = 0.34). These findings indicate that while ferritin significantly improved, erythropoiesis parameters (Hb and EPO requirement) remained largely unchanged, suggesting improved iron stores. A randomized controlled trial by Corrêa HL et al. (2021) [52] comparing two groups: a control group and a resistance training group, each further divided into three subgroups based on iron and erythropoietin treatment. It found that Hb levels increased in all exercise subgroups, indicating that exercise positively influenced anemia management. Serum ferritin levels decreased in the intervention group and the exercise group required less EPO compared to the control group. The discrepancies observed between the results of our study and the existing literature may be explained by the fact that the hemoglobin and ferritin levels of our participants were initially below the target values at baseline.

Body mass index (BMI) and body composition are key indicators of nutritional status and overall health in hemodialysis patients. Most of the available literature on intradialytic resistance exercise has studied the effect of this intervention on BMI and body composition.

Our study results showed a mild overall decrease in the participants’ BMI but this change was statistically not significant (p = 0.11). However positive shifts were noted in its distribution: Underweight category remained unchanged (n = 2), we also noted an increase in the healthy weight category (n = 12 to n = 13) and a reduction in the overweight category (n = 7 to n = 5). In line with our study findings, Kopple et al. [53] detected a reduction in BMI in all exercising groups irrespective of the type of training, (i.e., −0.3 m/kg2 in the endurance training group, −1.0 m/kg2 in the strength training group, and −0.2 m/kg2 in the combined group), whereas in the control group, BMI increased by 0.1 m/kg2 at the end of the intervention. Contrary to our study results, most of the reviewed literature especially two studies examining BMI shifts after different periods of intradialytic exercise, reported a slight increase. For instance, Baiao et al., 2023 [28] reported significant increases in BMI at four and eight months in patients undergoing intradialytic resistance training, compared to baseline measurements. Also, Kato et al., 2021 [35] concluded that one-year of intradialytic leg exercises with resistance bands resulted in an increased BMI among elderly hemodialysis patients. However, none of these two studies reported participants BMI distribution and category changes, also the differences found compared to our study results may be explained by the intervention duration. In fact, both studies lasted for at least eight months. It may also be explained by the exercise intensity and modality. Higher resistance loads may have led to greater muscle mass gain, which could explain the increase in BMI. That’s why most studies focused on the shifts in body composition metrics.

To assess body composition we made sure to take post-dialysis measurements in our study population to limit fluid shifts interference with metrics accuracy. At baseline our populations’ body composition metrics were as follow: A mean Total body Water (TW) of 51.7%, a mean fat Mass of 25.9% and a mean muscle Mass: 34.9%. While standard reference ranges for dialysis patients are not well defined due to fluid imbalances and other factors, our participants’ measurements fall roughly within normal range for general population.

After intervention period we noted that exercise did not significantly impact total weight or dry weight. But, fat mass decreased significantly by 2.8% (p = 0.003), and total body water increased significantly by 4.5% (p < 0.001). As for muscle mass we noted a slight, not statistically significant, decrease of 1.4% (p = 0.14). The effects of intradialytic exercise on body fat, and lean body mass varied among different studies. Michou et al., 2023 [22] showed that a 4-month intradialytic exercise program resulted in a 2.7% increase in lean mass, similarly to our study, it showed a 5% reduction in body fat and in an increase in total body water among hemodialysis patients awaiting kidney transplant. In the same way also Marinho et al. [50] reported a higher reduction in fat mass in the intradialytic exercise group compared to the controls (mean difference −0.9% versus −0.6%, respectively), however in this same study [50], they found that resistance exercise led to increased muscle mass compared to the initial measurement, but the increase was smaller in the intervention group compared to the controls (~1% versus 3%). In conclusion body composition metrics varied widely across different studies with results impacted mostly by intervention duration and exercise modality, explaining the differences observed in comparison with our study.

Although the Mini Nutritional Scale (MNA) assessment has been established as a useful routine screening tool for malnutrition risk in patients on maintenance dialysis, few studies have used it to assess the impact of intradialytic exercise on nutritional status. In fact, most studies have used geriatric assessment scales, which can be explained by the older mean age of the participants. In our study, 69.9% of our participants were at risk of malnutrition at baseline, while 4.8% were malnourished, these results align with Nasir et al. 2022 [19] concluding that 57.4% of the study participants were at risk of developing malnutrition while 4.6% of patients were malnourished. After intervention period we noted a decrease in the percentage of patients at risk of malnutrition (61.9% to 47.6%) while the malnourished category disappeared. Consistent with our study results, in the study by Frih et al, 2017 [54] in which the MNA was also performed, it was observed that the nutritional status improved after the intervention of resistance training, specifically, at baseline in the passive and intervention groups, the risk of malnutrition was 85.7% and 70% respectively, and at the final assessment the risk of malnutrition in the intervention group was 23.8% compared to a risk of 85% in the passive group.

One of the most important outcomes in exercise interventions for hemodialysis patients is adherence and consistent participation to exercise programs. In fact, adherence tends to be a core determinant for every other beneficial outcome including improvements in physical function, mental health, dialysis adequacy and all of the above-mentioned key points. However, implementing and sustaining it in clinical practice has proved to be challenging.

In our trial, the mean adherence rate to intradialytic resistance exercise was 63.8%, with a minimum of 10% and a maximum of 100%. A total of 15 patients (71.4%) maintained an adherence rate above 50%. Notably, no male patient had an adherence rate below 50%. The main identified reason of non adherence was patient’s refusal to exercise at given days. Compared to current literature, our results are slightly under the reported rates. For example, in a Large-Scale nationwide Implementation Study conducted by Martins et al., 2022 [27] across 21 dialysis unit in Portugal the reported adherence rate was 75.0% ± 19.7%. Non-performed sessions were mainly due to patient refusal (61.5%) and pain (8.4%). He also concluded that younger patients with better health status were more likely to participate. Likewise, a Feasibility study by Ribeiro et al., 2022 [34] has found a 79.6% adherence rate over 953 potential sessions, with the highest adherence (86.6%) in the initial weeks and a decline to 73.5% later on. Comparable adherence rates were reported by Chan et al., 2016 [55] with 11 of the 18 participants achieved at least 75% adherence, attending more than 27 of the 36 sessions offered during the 12-week intervention.

Adherence rates vary widely based on when and where the exercise is performed, we especially highlight the sustainability of intradialytic exercise vs interdialytic or at-home exercise, hence we consolidate our choice of this exercise modality. In fact, patients prefer to make use of intradialytic time instead of sparing extra time for exercise, also the healthcare providers’ supervision increases motivation and safety reducing fear of injury or complication [21]. Moreover, the encouragement from dialysis staff plays a key role in maintaining adherence. However, no significant associations were found between intervention characteristics (exercise type, duration, frequency and setting) and adherence as evidenced by the scoping review published by Agarwal et al., 2025 [56] analyzing 55 randomized controlled trials involving 3,269 participants.

There are many hurdles to the successful implementation of an intradialytic exercise protocol. These barriers fall into three main categories: staff-related, patient-related and protocol-related factors. In a descriptive study untitled “Motivation, Barriers, and Suggestions for Intradialytic Exercise—A Qualitative Study among Patients and Nurses”, Wodskou et al. 2021 [57] reported that dialysis unit nurses had a pre-conceived safety concern about Intradialytic exercise such us vascular access site removal. They also expressed concerns about increased workload and time constraints and suggested that exercise professionals should supervise intradialytic exercise rather than dialysis staff. Potential participants also expressed concerns about triggering dialysis machine alarms and disturbing nurses.

Concerns about exercise protocol included mostly lack of staff expertise, limited unit personnel and limited resources and especially the absence of a standardized exercise protocol, as highlighted by the trial conducted by Valenzuela et al. 2018 [58].

Although our study adherence rate was slightly lower than that found in literature, our results were overall satisfactory. We hereby, emphasize on the feasibility and low cost of our intervention. The cost-effectiveness of intradialytic exercise has been thoroughly assessed in current literature and it depends on several factors, including the type of equipments used, the level of supervision, and the duration of the intervention. In aerobic exercise setting for example, the PEDAL trial [59] reported that the cost of delivering a 6-month intradialytic exercise program ranged from £463 to £848 per participant per year. However, resistance training programs using elastic bands, similar to our trial, or ankle weights have been shown to be more cost effective, with minimal equipment requirements [60,61].

The safety of intradialytic resistance exercise has been consistently demonstrated in trials. In our study, we reported mild adverse events in two patients, one episode of hypertension and one patient experiencing muscle cramps. We note that these events occur in dialysis patients independently of intradialytic exercise or any other intervention. Our study joins the randomised controlled trial by Martins et al, 2022 [27] which found no significant differences in adverse events between the exercise and non-exercise groups. Ribeiro et al, 2022 [34] also reported no serious complications during resistance training, with only minor problems such as hypotension. These findings underline the safety of intra-dialytic resistance exercise, especially when properly supervised.

### Strenghts

- Our study addressed the trending strategy in dialysis patients’ care of intradialytic exercise.
- It studied the effect of this intervention on our north African HD population, helping to bridge a knowledge gap in underrepresented Non-Western, middle to low income regions.
- We provided a holistic assessment of intradialytic exercise benefits addressing both effects on physical capacity and also evaluating mental health and quality of life.
- We acknowledged the main challenge: adherence variability and we highlighted its different barriers.
- We showed that it is a Low-Cost, Easy-to-Implement Strategy with Potential for Clinical Integration, proving its accessibility for dialysis centers with limited resources.
- Hence, it is a real-world study that proved to be safe and feasible

### Limitations

- The absence of a control group makes it difficult to definitively attribute improvements solely to intradialytic exercise.
- The small study sample and the fact that our study is a single center trial may limit the generalizability of our findings.
- Although the study duration was sufficient to establish significant improvements but the lack of long term follow up may limit long-term sustainability
- Although we offered our participants different elastic bands intensities based on individual tolerance and preference, we did not systematically track exercise intensity and progression.
- Although all assessments were carried out under similar procedural conditions, some environmental factors such as temperature and atmospheric pressure were not controllable by the experimenters. These variations may have introduced minor differences in test performance or physiological responses among participants and should therefore be considered when interpreting the results.

### Recommendations

#### Standardization of exercise protocols and integration into dialysis routine.

We recommend developing a standardized intradialytic exercise program combining resistance and aerobic components and to creating training manuals and videos for dialysis staff to uniformly apply the exercise protocols.

Giving its demonstrated feasibility and safety we recommend implementing the intradialytic exercise as a standard practice in dialysis care.

#### Patient engagement and motivation.

We should educate patients on the benefits of intradialytic exercise via flyers, videos, and testimonials and seek to implement simple motivation tools like progress tracking sheets, group sessions, or patient ambassadors.

#### Capacity building.

Organize **training workshops** for nephrologists, nurses, and physiotherapists across Tunisia and seek partnerships with universities (e.g., sports science faculties) to involve interns and master’s students in program supervision and research.

## Conclusion

We conducted a single-center, pre-experimental clinical trial over a 3-month period to evaluate the effects of supervised intradialytic resistance exercise in patients undergoing maintenance hemodialysis. This study represents the first of its kind in Tunisia and North Africa and is the result of a unique and successful collaboration between nephrologists, physiotherapists, and dialysis nurses—underscoring the importance of interdisciplinary care in managing chronic kidney disease. Our findings demonstrate that such collaborative, non-pharmacological interventions can meaningfully improve physical function, dialysis adequacy, and nutritional status. Importantly, the intervention proved to be safe, feasible, and well-tolerated, even within a resource-limited setting. Further randomized controlled trials are warranted to validate these findings and support broader clinical implementation.

## Supporting information

S1 File Base Rev. (XLSX)

S2 File cdc_149677_DS1. (DOCX)

S3 File Clinical Trial Protocol supplementary files. (DOCX)

S4 File Research protocol. (PDF)
