# Supplementary material for: The effect of intradialytic resistance exercise on physical function and dialysis adequacy in patients on maintenance hemodialysis
Source: PLoS One. 2026 Mar 13;21(3):e0337910. doi: 10.1371/journal.pone.0337910 (PMC12987497; doi:10.1371/journal.pone.0337910)
Supplement: S2 File — (DOCX) [file pone.0337910.s002.docx]

| **Paper Section/**  **Topic** | **Item No** | **Descriptor** | **Reported?** | |
| --- | --- | --- | --- | --- |
|  |  |  | 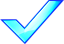 | **Pg#** |
| **Titleand Abstract** | | | | |
| Title and Abstract | 1 | 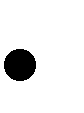Information on how unit were allocated to interventions | 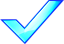 | 1-2 |
|  |  | 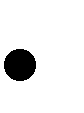Structured abstract recommended | 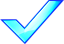 | 1-2 |
|  |  | 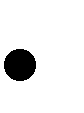Information on target population or study sample | 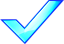 | 1-2 |
| **Introduction** | | | | |
| Background | 2 | 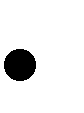Scientific background and explanation of rationale | 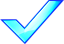 | 2-3 |
|  |  | 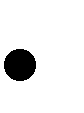Theories used in designing behavioral interventions |  | 2-3 |
| **Methods** | | | | |
| Participants | 3 | 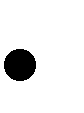Eligibilitycriteriaforparticipants,includingcriteriaatdifferentlevelsin recruitment/sampling plan (e.g., cities, clinics, subjects) | 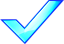 | 4-5 |
|  |  | Methodofrecruitment(e.g.,referral,self-selection),includingthe sampling method if a systematic sampling plan was implemented 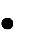 | 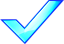 | 4-5 |
|  |  | Recruitmentsetting 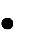 | 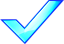 | 4-5 |
|  |  | Settingsandlocationswherethedatawerecollected 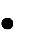 | 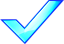 | 4-5 |
| Interventions | 4 | 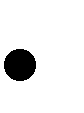Detailsoftheinterventionsintendedforeachstudyconditionandhow and when they were actually administered, specifically including: | 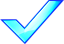 | 5-7 |
|  |  | - Content:whatwas given? | 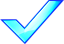 | 5-7 |
|  |  | - Deliverymethod:howwasthecontentgiven? | 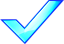 | 5-7 |
|  |  | - Unitofdelivery:howwerethesubjectsgroupedduringdelivery? | 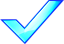 | 5-7 |
|  |  | - Deliverer:whodeliveredtheintervention? | 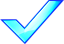 | 5-7 |
|  |  | - Setting:wherewastheinterventiondelivered? | 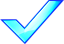 | 5-7 |
|  |  | - Exposurequantityandduration:howmanysessionsorepisodesor events were intended to be delivered? How long were they   intendedtolast? | 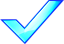 | 5-7 |
|  |  | - Timespan:howlongwasitintendedtotaketodeliverthe   interventiontoeach unit? | 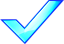 | 5-7 |
|  |  | - Activitiestoincreasecomplianceoradherence(e.g.,incentives) | 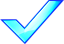 | 5-7 |
| Objectives | 5 | 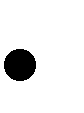Specific objectives and hypotheses | 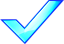 | 7 |
| Outcomes | 6 | 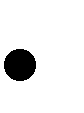Clearly defined primary and secondary outcome measures | 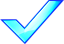 | 7 |
|  |  | 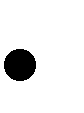Methodsusedtocollectdataandanymethodsusedtoenhancethe quality of measurements | 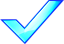 | 7 |
|  |  | 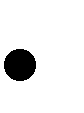Informationonvalidatedinstrumentssuchaspsychometricand biometric properties | 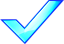 | 7 |
| SampleSize | 7 | Howsamplesizewasdeterminedand,whenapplicable,explanationofany interim analyses and stopping rules 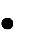 | 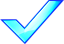 | 5 |
| Assignment Method | 8 | Unitofassignment(theunitbeingassignedtostudycondition,e.g., individual, group, community) 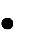 |  |  |
|  |  | Methodusedtoassignunitstostudyconditions,includingdetailsofany restriction (e.g., blocking, stratification, minimization) 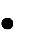 |  |  |
|  |  | Inclusionofaspectsemployedtohelpminimizepotentialbiasinduceddue to non-randomization (e.g., matching) 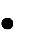 |  |  |

| Blinding (masking) | 9 | 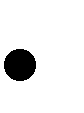Whether or not participants, those administering the interventions, and thoseassessingtheoutcomeswereblindedtostudyconditionassignment; if so, statement regarding how the blinding was accomplished and how it was assessed. |  |  |
| --- | --- | --- | --- | --- |
| UnitofAnalysis | 10 | 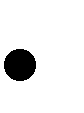Descriptionofthesmallestunitthatisbeinganalyzedtoassess intervention effects (e.g., individual, group, or community) | 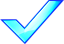 | 7-9 |
|  |  | 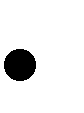Iftheunitofanalysisdiffersfromtheunitofassignment,theanalytical method used to account for this (e.g., adjusting the standard error estimates by the design effect or using multilevel analysis) | 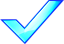 | 7-9 |
| Statistical Methods | 11 | 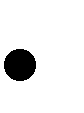Statisticalmethodsusedtocomparestudygroupsforprimarymethods outcome(s), including complex methods of correlated data | 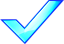 | 10 |
|  |  | 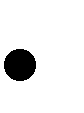Statisticalmethodsusedforadditionalanalyses,suchasasubgroup analyses and adjusted analysis |  |  |
|  |  | Methodsforimputingmissingdata,if used 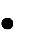 |  |  |
|  |  | Statisticalsoftwareorprogramsused 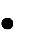 | 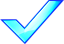 | 10 |
| **Results** | | | | |
| Participantflow | 12 | Flow of participants through each stage of the study: enrollment, assignment,allocation,andinterventionexposure,follow-up,analysis(a diagram is strongly recommended) 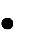 | 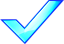 | 10 |
|  |  | - Enrollment:thenumbersofparticipantsscreenedforeligibility, found tobeeligible ornoteligible, declinedto beenrolled, and   enrolledinthestudy | 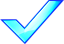 | 10 |
|  |  | - Assignment:thenumbersofparticipantsassignedtoastudy   condition | 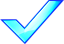 | 10 |
|  |  | - Allocationandinterventionexposure:thenumberofparticipants assigned to each study condition and the number of participants   whoreceivedeachintervention | 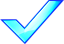 | 10 |
|  |  | - Follow-up:thenumberofparticipantswhocompletedthefollow- up or did not complete the follow-up (i.e., lost to follow-up), by   studycondition | 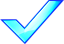 | 10 |
|  |  | - Analysis:thenumberofparticipantsincludedinorexcluded from   themainanalysis,bystudycondition | 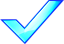 | 10 |
|  |  | 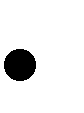Descriptionofprotocoldeviationsfromstudyasplanned,alongwith reasons |  |  |
| Recruitment | 13 | Datesdefiningtheperiodsofrecruitmentandfollow-up 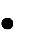 | 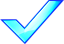 | 5 |
| BaselineData | 14 | Baselinedemographicandclinicalcharacteristicsofparticipantsineach study condition 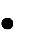 | 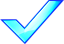 | 11-13 |
|  |  | Baselinecharacteristicsforeachstudyconditionrelevanttospecific disease prevention research 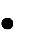 | 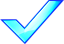 | 11-13 |
|  |  | Baselinecomparisonsofthoselosttofollow-upandthoseretained,overall and by study condition 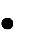 | 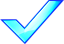 | 11-13 |
|  |  | Comparisonbetweenstudypopulationatbaselineandtargetpopulation of interest 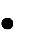 | 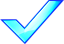 | 11-13 |
| Baseline equivalence | 15 | 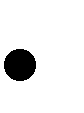Dataonstudygroupequivalenceatbaselineandstatisticalmethodsused to control for baseline differences | 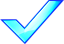 | 11-13 |

| Numbers analyzed | 16 | 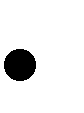Number of participants (denominator) included in each analysis for each studycondition,particularlywhenthedenominatorschangefordifferent  outcomes;statementoftheresultsinabsolutenumberswhenfeasible | 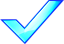 | 11-13 |
| --- | --- | --- | --- | --- |
|  |  | 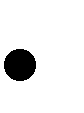Indicationofwhethertheanalysisstrategywas“intentiontotreat”or,if not, description of how non-compliers were treated in the analyses |  |  |
| Outcomesand estimation | 17 | 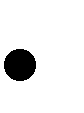For each primary and secondary outcome, a summary of results for each estimationstudycondition,andtheestimatedeffectsizeandaconfidence interval to indicate the precision | 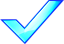 | 13-18 |
|  |  | 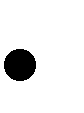Inclusion of null and negative findings | 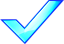 | 13-18 |
|  |  | Inclusionofresultsfromtestingpre-specifiedcausalpathwaysthrough which the intervention was intended to operate, if any 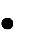 | 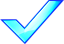 | 13-18 |
| Ancillary  analyses | 18 | 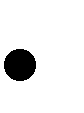Summaryofotheranalysesperformed,includingsubgrouporrestricted analyses, indicating which are pre-specified or exploratory |  |  |
| Adverseevents | 19 | 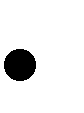Summary of all important adverse events or unintended effects in each studycondition(includingsummarymeasures,effectsizeestimates,and  confidenceintervals) |  | 18 |
| **DISCUSSION** | | | | |
| Interpretation | 20 | 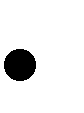Interpretation of the results, taking into account study hypotheses,sourcesofpotentialbias,imprecisionofmeasures,multiplicativeanalyses,  andotherlimitationsorweaknessesofthestudy | 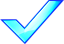 | 18-30 |
|  |  | Discussionofresultstakingintoaccountthemechanismbywhichthe intervention was intended to work (causal pathways) or alternative mechanisms or explanations 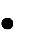 | 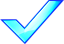 | 18-30 |
|  |  | Discussionofthesuccessofandbarrierstoimplementingtheintervention, fidelity of implementation 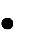 | 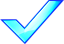 | 18-30 |
|  |  | Discussionofresearch,programmatic,orpolicyimplications 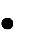 | 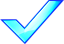 | 18-30 |
| Generalizability | 21 | 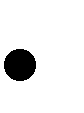Generalizability(externalvalidity)ofthetrialfindings,takingintoaccount the study population, the characteristics of the intervention, length of follow-up, incentives,compliance rates, specific sites/settings involved in  thestudy,andothercontextualissues | 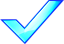 | 18-30 |
| Overall  Evidence | 22 | 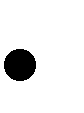Generalinterpretationoftheresultsinthecontextofcurrentevidence and current theory | 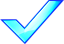 | 30-31 |

*From:*Des Jarlais, D. C., Lyles, C., Crepaz, N., & the Trend Group (2004). Improving the reporting quality of nonrandomizedevaluationsofbehavioralandpublichealthinterventions:TheTRENDstatement.*AmericanJournalof Public Health*, 94, 361-366.For more information, visit: <http://www.cdc.gov/trendstatement/>
