# Supplementary figures and images for: The effect of intradialytic resistance exercise on physical function and dialysis adequacy in patients on maintenance hemodialysis
Source: PLoS One. 2026 Mar 13;21(3):e0337910. doi: 10.1371/journal.pone.0337910 (PMC12987497; doi:10.1371/journal.pone.0337910)

**Information Letter**


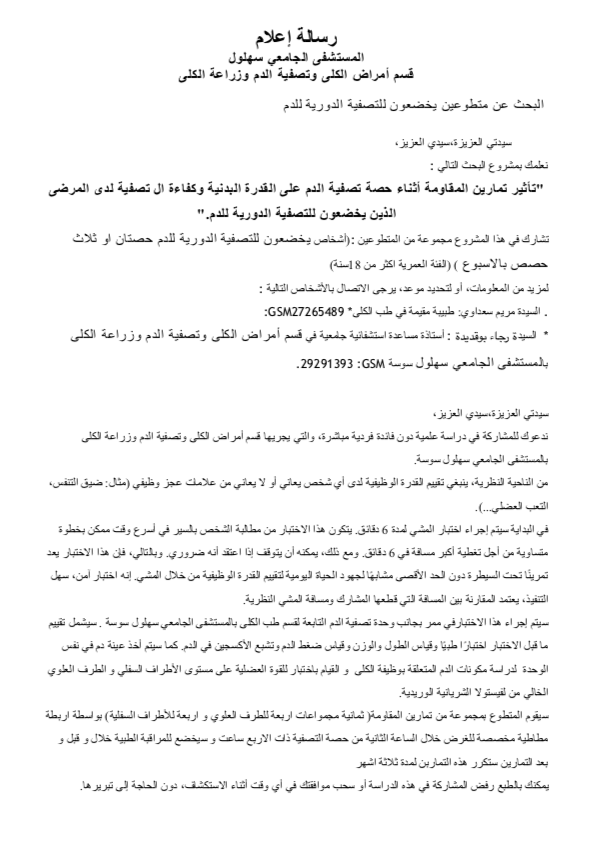


Consent Forms


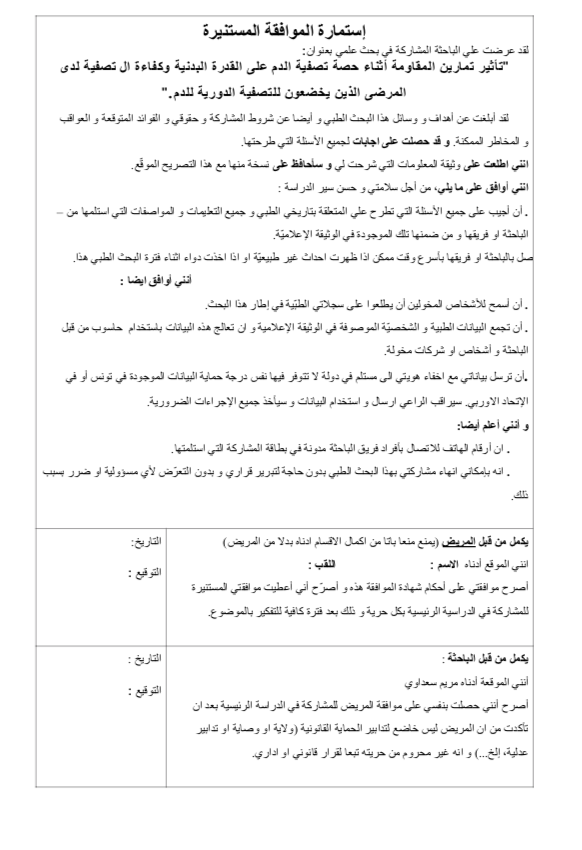


Patients informations forms


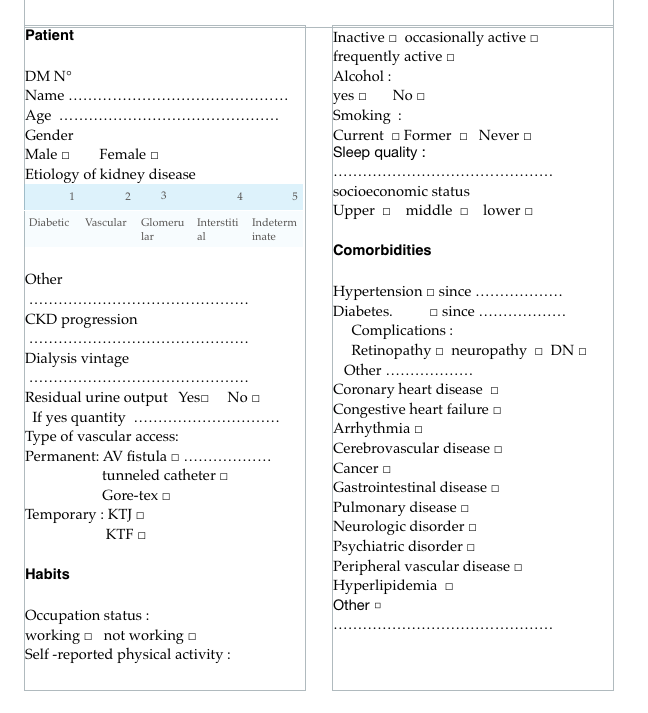


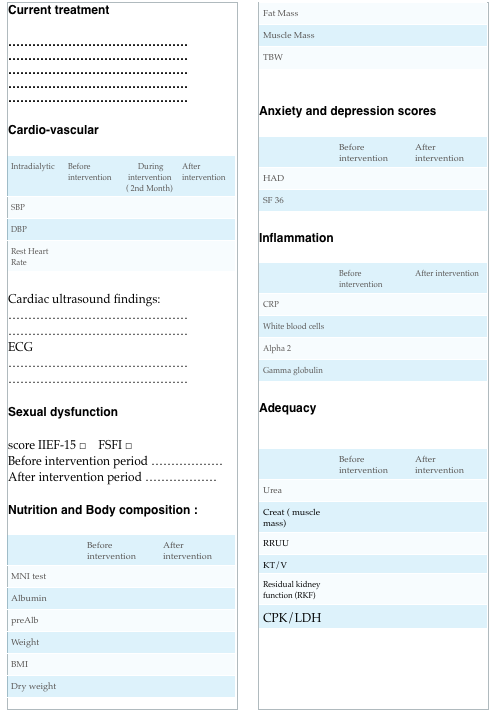

Supplement: S3 File — (DOCX) [file pone.0337910.s003.docx]
