# Supplementary material for: The effect of intradialytic resistance exercise on physical function and dialysis adequacy in patients on maintenance hemodialysis
Source: PLoS One. 2026 Mar 13;21(3):e0337910. doi: 10.1371/journal.pone.0337910 (PMC12987497; doi:10.1371/journal.pone.0337910)
Supplement: S4 File — (PDF) [file pone.0337910.s004.pdf]

The Effect of intradialytic resistance exercise on physical function and Dialysis adequacy in patients on maintenance hemodialysis

Resarch Protocol

Nephrology Department - University Hospital of Sahloul

Raja Boukadida

Mariem Saadaoui

I. Background/ Research question:

Chronic kidney disease (CKD) is a progressive condition that affects more than 10% of the general population worldwide, amounting to more than 800 million individuals (1).

End-stage renal disease (ESRD), the last stage of CKD, is an emerging global public health problem and it requires patients to undergo regular renal replacement therapy (RRT). Dialysis is the predominant RRT in most countries, with maintenance hemodialysis (HD) being the most common modality.

In 2008 the prevalence of patients undergoing HD was 122 per million population worldwide and 750 per million population in Tunisia and its incidence is growing by approximately 8% annually (2,3).

Progressive CKD is linked to several complications with higher prevalence and intensity in HD population, which interact with each other (4) :

- Hypertension.
- Cardiovascular complications.
- Anemia.
- CKD-related mineral bone disorder.
- Salt and water retention.
- Metabolic acidosis and electrolyte disorders.
- Uremic syndrome.
- Poor nutritional status and skeletal muscle loss(5).

These complications contribute to high morbidity and mortality and poor quality of life and were identified as being relevant to the global burden of poor health caused by CKD (4).

Other complications are well established but difficult to assess thus understudied :

- The loss of physical function, associated or exacerbated by the high prevalence of coexisting comormobdities (6).
- Depression with an estimated incidence of 20 to 30% (7), second only to hypertension in frequency as a comorbid diagnosis in patients with ESRD (8,9) as well as an altered quality of life.

Despite the current substantial medical and pharmacological advances, and giving the complications above, HD patients continue to have strikingly higher mortality rates compared to age, gender, and race-matched populations and the most important predictors of poor outcome are increasing age, cardiovascular disease, diabetes, and poor nutrition(5) .Higher mortality risk has been reported for sedentary patients (6).

In recent years, the potential of physical exercise as a therapeutic tool in patients with ESRD undergoing maintenance HD treatment has been investigated and research suggested that Increasing activity levels is a promising solution to combat muscle wastage(6,10,11), as it increases dialysis adequacy (12, 13, 14) and other clinically relevant outcomes such as patients' functional capacity and quality of life(15).

Intradialytic exercise (16), defined as any type of exercise performed during dialysis sessions has been proposed as a viable strategy to increase physical activity in this population.

As previous studies have suggested that intradialytic exercise is effective in enhancing exercise tolerance, improving quality of life and psychological status. Research also indicates that intradialytic exercise can increase the efficacy of dialysis, subsequently alleviating inflammation, improving nutrition and bone mineral density.

The effect of intradialytic training interventions published to date have been modest on our Tunisian population with it’s demographic and cultural specificities that’s why our project aims to :

- Determine the impact of intradialytic exercise in the improvement of functional and metabolic status in HD patients.
- Examine the effects of an intradialytic exercise program on physical performance, and quality of life in HD patients.
- Evaluate the safety of intradialytic exercise, as well as its effects, in terms of maintenance HD patient’s clinical outcomes.

II. Literature review

With an increasing prevalence, renal replacement therapy especially maintenance HD for ESRD is a worldwide concern due to rising health care costs.

Multiple studies had underlined that physical activity is exceptionally low amongst the HD population. A prospective controlled study (17) conducted in 2005 had observed that elderly dialysis patients have a 50% lower functional capacity compared to healthy individuals, regardless of age and gender. Other reviews underlined inactivity as a powerful predictor of ***mortality***. In a systematic review (18) published in BMC nephrology in 2021 by Martins et al. Including eleven studies: six in hemodialysis, three in kidney transplant, and two in hemodialysis and peritoneal dialysis patients held in four different continents, a number of participants ranging from 109 patients to 20920 patients was reviewed . This review had addressed

the effect of intradialytic exercise on all cause and cardiovascular mortality in end-stage renal disease patients. Nine studies concluded to a significant reduction in all cause mortality with increased levels of physical activity and evidence of a dose-response relationship. For cardiovascular mortality, a significant reduction was observed in two of the three studies. Other observational studies involving different cohorts (6) (10) have also reported that increased physical activity level was not only associated with decreased mortality among hemodialysis patients but also an effective approach to improve other clinically relevant outcomes in these patients.

Intradialytic exercise has been studied as an effective use of the intradialytic period and to incorporate physical activity in these patients under professional supervision. An umbrella review of systematic reviews and/or meta-analytical studies (19) including eleven reviews and 48 unique meta-analyses had examined intradialytic exercise regardless of its intensity, frequency, duration, and the moment of exercise during the dialysis session in adults patients on hemodialysis . It had provided a general overview and appraisal of the effects of different intradialytic exercise modalities showing Clinical benefits for functional capacity associated with both aerobic exercise and resistance , measured by the distance covered in the 6-minutes walking test. It had also shown that exercise may reduce fatigue through mechanisms related to the anti-inflammatory effects of physical activity therefore has a positive effect on patients's quality of life.

Although ***Tunisia*** has the highest prevalence of hemodialysis patients compared to the other countries in North Africa (2)

studies eveluating the effect of intradialytic training interventions published to date have been modest to non existent. In 2017 Frih et al. (20) conducted a single blinded, randomized, controlled study of interdialytic combined endurance-resistance training program including 41 patients concluding to significant improvement in physical performance assessed by the 6-min walk test, improved nutritional status, significantly decreased depression scale and to an improvement in systolic blood pressure. Another prospective controlled trial (21) conducted in 2022 by Marzougui et al. confirmed that Intradialytic concurrent training induced beneficial effects on physical function, muscle strength, postural balance, and quality of life assessment scales in HD patients.

Exercise timing and modalities: Both types of aerobic and resistance exercises can be done by the patients as complementary and practical, affordable, and available therapy with similar effectivness. Intradialytic aerobic exercise has been shown to be safe in the first 2 hours of dialysis; after 2 hours, to improve peak oxygen consumption and quality of life. Storer et al (22) showed that intradialytic cycling increases peak oxygen consumption, power, endurance time, and quadriceps strength and improves fatigability. Dialysis efficacy and physical functioning improve with intradialytic cycle ergometer exercise. Resistance training also improves exercise capacity. Diesel and colleagues (23) showed a stronger correlation between indices of muscular strength and exercise capacity than between variables that reflect oxygen-carrying capacity and exercise tolerance in ESRD patients. Although the best protocol of exercise for patients on dialysis is not yet clear, current literature promotes the combination of aerobic and resistance training as more effective than

resistance alone or aerobic training to improve functional performance. A duration of 8 weeks with 3 exercise sessions a week corresponding with patient's thrice-weekly dialysis schedule has been the modality chosen by most of the RCT reviewed. The prescribed exercise duration was 60 minutes, performed as two 30-minute exercise bouts with a 30-minute recovery period between bouts during the first 2 hours of a 4-hour dialysis session. In fact previous investigations showed that three 15-minute bouts of exercise during HD were insufficient to cause a detectable increase in serum urea removal and that two 30-minute bouts of exercise substantially elevated the amount of urea removed in dialysate fluid.

According to current literature The 6-minutes walking test (6MWT) seems to be the best option for assessing ***Functional capacity*** (18 , 19, 20, 21): The meta-analyses and randomized controlled trials reviewed reported assessing the 6MWT as it is easy to apply, less expensive, more tolerable, and more representative of the efforts HD patients make in activities of daily living.It is also a predictor of all-cause mortality and it is associated with cardiorespiratory fitness and endurance. The results of the meta-analysis (22) published in 2019 including 27 studies with a total of 1,156 participants for the 6MWT indicate that overall exercise, regardless of modality and timing of delivery, improves distance walked on the 6MWT at a mean of 33.64 m. VO2 peak was measured in some studies . However, the incremental effort required by this test causes strong metabolic stress makes it less tolerable. Therefore, it can be difficult for patients with an impaired functional capacity which is the case for most patients with end-stage renal disease.

Research also indicates that intradialytic exercise can increase the ***Dialysis adequacy:*** A controlled trial (12) including 11 HD patients who underwent aerobic intradialytic exercise had suggested improvment in $Kt/V_{urea,}$ of serum urea clearance (UPR) (23) as well as a reduction in rebound ratios of creatinine, phosphate and β_2-microglobulin. A study (24) published in 2019 by Jiang Pu et al. reviewing a total of 27 RCTs involving 1215 subjects showed that intradialytic exercise could improve Kt/V . ***Depression and quality of life*** were reviewed in four

RCTs involving 195 patients using Hospital Anxiety and Depression Scale (HAD) and the Medical Outcomes Study Short Form 36-Item (SF-36). The results showed that intradialytic exercise was able to lower the depression level and to improve two aspects of quality of life short form questionnaire. Results concerning the effect of intradialytic exercise on ***Inflammation*** were not consistent: some studies have shown the positive effect of exercise in reducing the inflammatory state of HD patients, achieving better benefits with long duration programs with a higher intensity. However, other studies showed no effect on inflammation biomarkers, such as CRP, IL-6 or TNFα which calls for caution in drawing strong conclusions to avoid overestimating its effect (25). Reviews always studied the effect of intradialytic resistance exercise on phosphorus, anemia and erythropoitin need but resuls were conflicting.

The reviewed studies consistently demonstrated that any modality of intradialytic exercise in this case resistance exercice has positive effects on physical function and dialysis adequacy

and other clincally relevant outcomes in patients on maintenance hemodialysis.

III. Key definitions:

❖ Chronic Kidney Disease (CKD):

CKD is defined as kidney damage or glomerular filtration rate (GFR) <60 mL/min/1.73 m2 for 3 months or more, irrespective of cause. Kidney damage in many kidney diseases can be ascertained by the presence of albuminuria, defined as albumin-to-creatinine ratio >30 mg/g in two of three spot urine specimens. GFR can be estimated from calibrated serum creatinine and estimating equations, such as the Modification of Diet in Renal Disease (MDRD) Study equation or the Cockcroft-Gault formula. Kidney disease severity is classified into five stages according to the level of GFR.

❖ ESRD (End Stage Renal disease) : It is stage 5 of chronic

Stage	GFR (ml/min/1.73m^2)	Terms
1	≥90	Normal or high
2	60-89	Mildly decreased
3a	45-59	Mildly to moderately decreased
3b	30-44	Moderately to severed decreased
4	15-29	Severly decreased
5	<15	Kidney failure

kidney disease.

❖ Six minute Walk test :

Patients walked a premeasured, outdoor circuit (35m), attempting to cover as much distance as they could in 6 minutes, with the measured distance in meters as the outcome. Blood pressure and heart rate were determined at rest and 6 minutes (peak exercise).

❖ Urea Clearance (UPR):

Urea is a middle molecular weight molecule that readily crosses the dialysis membrane and the clearance of this molecule from the blood during dialysis is the standard measure of dialysis efficacy. urea clearance was determined at baseline and at the end of the program.

❖ KT/V:

SpKt/V is a dimensionless value representing fractional urea clearance and reflects the exchange of urea across an interface; the vascular compartment and the dialyser. The minimum target dose (in spKt/V) recommended by the Kidney Disease Quality Outcomes Initiative is 1.2. in Our study it is calculated by the simplified Equations of Daugirdas (27).

❖ Medical Outcomes Study Short Form 36-Item (SF-36):

This questionnaire consists of 36 items compiled into 8 scales: physical functioning (PF), role functioning/physical (RP), bodily pain (BP), general health (GH), vitality (VT), social functioning (SF), role functioning/emotional (RE), and mental health (MH). These scales range from 0 to 100; a higher score is more positive it was administered at baseline and at the end of the exercise program.

❖ Hospital Anxiety and Depression Scale (HAD)

The HADS was developed to assess both anxiety and depression. This questionnaire consists of an anxiety symptoms subscale and a depressive symptoms subscale It has been found to be a valid assessment of the risk in primary care and it was administered at baseline and at the end of the exercise program.

❖ The mini nutritional assessment (MNA) :

It consists of a screening part and an assessment part**.** The screening part contains 6 items concerning decline of food intake, weight loss in the past 3 months, acute mobility, disease/ distress, neuropsychological problems, and additional anthropometric measures.The MNA test was administered at baseline and at the end of the exercise program.

IV. Materials and Methods

I. Study Design

Quasi-experimental 3-months clinical trial with supervised intervention of intradialytic resistance exercise training.

II. Study population

This study was conducted between May and October 2022 including 21 patients among 90 patients undergoing maintenance HD therapy at the Chronic Hemodialysis Unit in Sahloul University Hospital of Sousse.

1. Study sample
2. Selection process

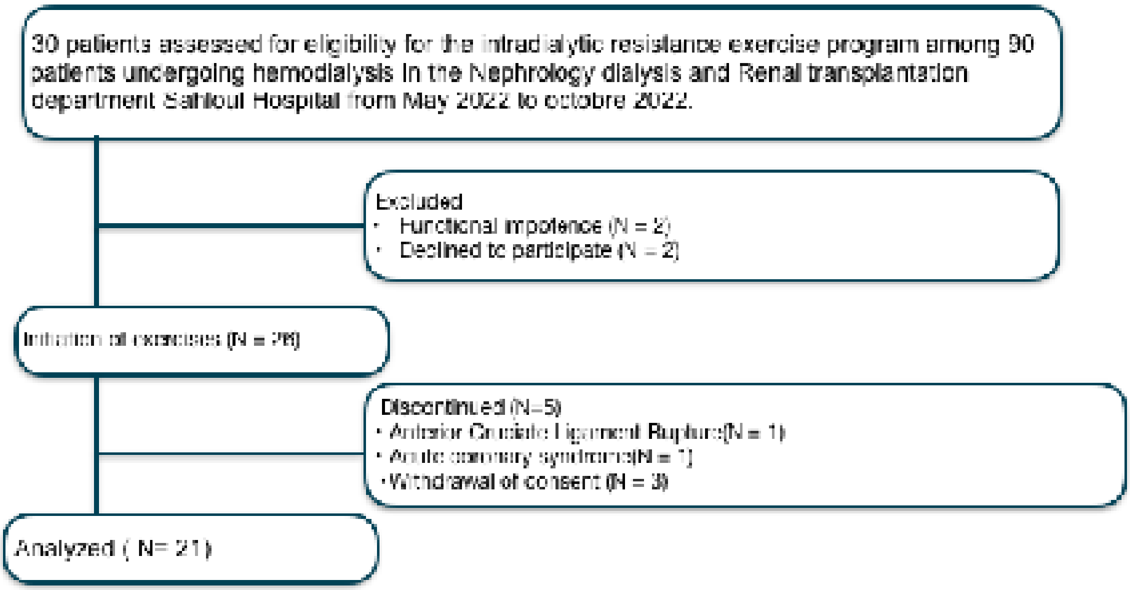

3. Inclusion Criteria :

- Adult patients of both sexes .
- Undergoing Maintenance HD for at least three months at least twice a week.
- Patients who voluntarily gave a written informed consent.

4. Exclusion Criteria :

- History of myocardial ischemia in the last 6 months.
- Decompensated heart failure(New York Heart Association stage ≥ 3), symptomatic cardiovascular disease with functional disability.
- Uncontrolled arrhythmia,
- Uncontrolled hypertension defined as systolic blood pressure (SBP) ≥ 200 mmHg and/or diastolic blood pressure (DBP) ≥ 120 mmHg.
- Severe lung disease.
- Acute systemic infection.

- Severe renal osteodystrophy and/Or Musculoskeletal disor- ders preventing the patients from performing the exercises.

III. Methods:

1. Data Collection

Demographic characteristics and Patients medical history were collected at baseline. Blood samples for laboratory data were obtained from arteriovenous shunt just before starting the first hemodialysis session of the week at baseline and after in- tervention period.

Intradialytic clinical parameters were collected from dialysis charts.

Dry weight was determined by considering cardiothoracic ratio, blood pressure, clinical symptoms, and physical findings such as oedema. Body composition data and weight were recorded using an an impedance scale immediately at the end of the dialysis session at baseline and after intervention period.

Depression and anxiety were evaluated using Hospital Anxiety and Depression Scale (HAD) and quality of life was evaluated using Medical Outcomes Study Short Form 36-Item question- naire SF36 (see Appendix).

2. Study Procedure

3.1. General measures

- The exercise was undertaken at least twice a week, and the whole process lasted at least 12 weeks.
- The prescribed exercise duration was at least 30 minutes, performed the first 2 hours of a 4-hour dialysis session.
- This exercise protocol was chosen for 2 reasons, first, previous inves- tigations showed that 15-minutes of exercise during HD were insuffi- cient to cause a detectable increase in serum urea removal, second,

many HD patients were unable to exercise during the third hour of dialysis due to hypotension and fatigue.

- Patients' Blood pressure (BP) and heart rate (HR) measurement were evaluated before and during exercise (Canceled if systolic BP > 200 mmH, diastolic BP >110 mmHg or HR >120 beats per minute).

3.2. Resistance exercise Protocol:

A typical program consisted of eight lower and upper body (Free Hand with no vascular access) resistance exercises using thick-coloured elastic bands (Theraband) commonly used in physical therapy resistance programs.
We disposed of two different resistance Therabands : Medium (3 to 4 kg) and High intensity (4 to 6 kg) adapted to patients tolerance.

❖ Upper Limb

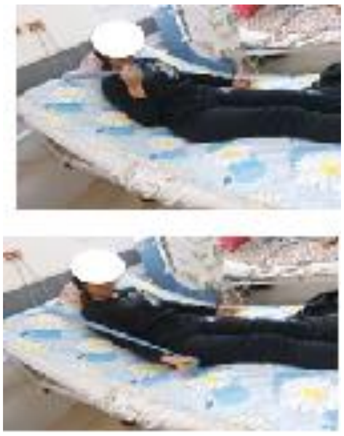

Three sets of 10 repetitions of medium intensity **Elbow extension** then adapted to patient's toler-

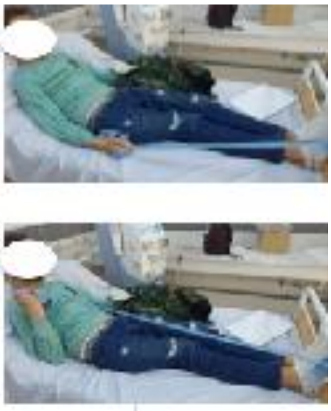

Three sets of 10 repetitions of medium intensity **Elbow flexion** then adapted to pa-

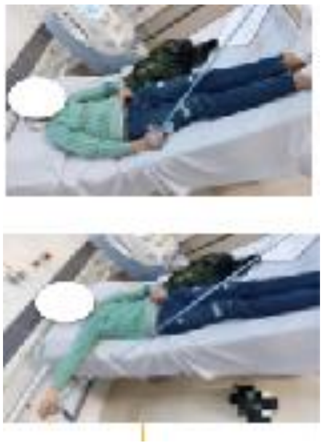

Three sets of 10 repetitions of medium intensity **Shoulder abduction** then adapted to patient's

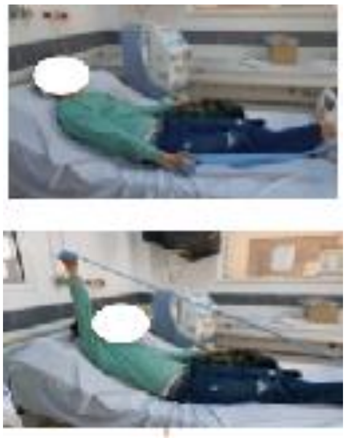

Three sets of 10 repetitions of medium intensity **Shoulder flexion** then adapted to patient's toler-

❖ Lower Limbs

3. Statistical Considerations and Data Analysis

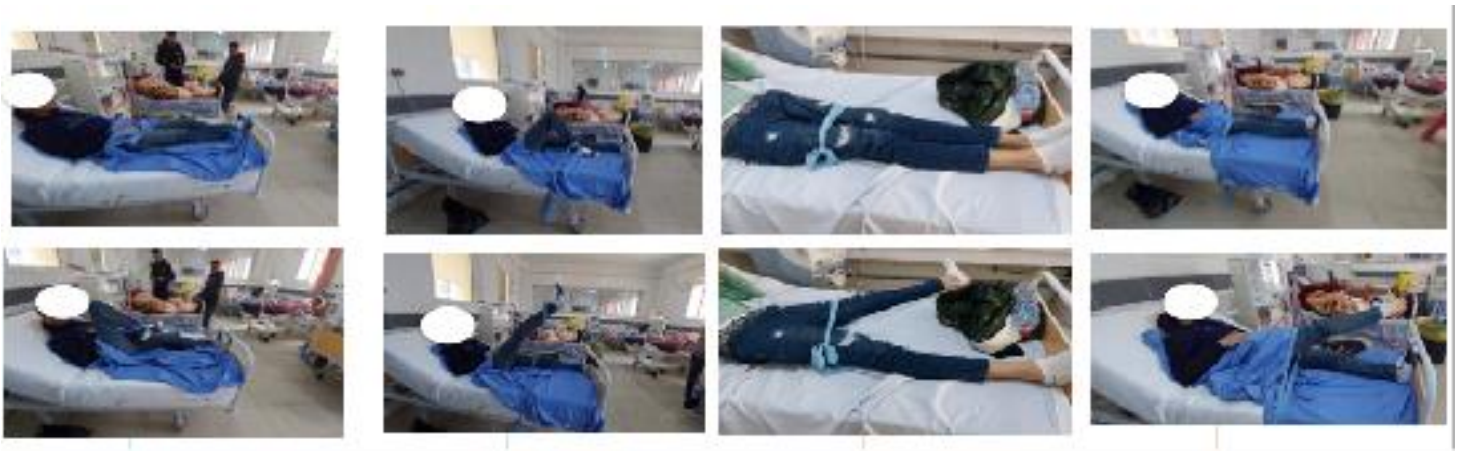

Quantitative variables were expressed as mean ± standard deviation and Categorical variables were summarized with percentages. The changes were analyzed using paired *t* test.

In all tests, *p* values of less than 0.05 were considered statistically significant.

All statistical analyses were performed using the IBM SPSS version 27.0

Bibliographie

1. Csaba P.Kovesdy. Epidemiology of chronic kidney disease: an update 2022 ,Kidney International Supplements (2022) 12, 7–11.
2. Vianda S. Stel et al. The 2008 ERA–EDTA Registry Annual Report- a précis NDT Plus (2011) 4: 1–13.
3. Schieppati A, Remuzzi G. Chronic renal disease as a public health problem: epidemiology, social, and economic implications. Kidney Int Suppl 2005;68 S98;7-10.
4. AK Bello et al. Enhancing optimal care for CKD-related complications, Kidney International Supplements (2017) 7, 122–129.
5. Saran R, Robinson B, Abbott KC, et al. US Renal Data System 2017 Annual Data Report: epidemiology of kidney disease in the United States. *Am J Kidney Dis*. 2018;71(3s1):A7
6. Ann M. O'Hare et . Dcreased Survival Among Sedentary Patients Undergoing Dialysis: Results From the Dialysis Morbidity and Mortality Study Wave 2 , *American Journal of Kidney Diseases,* Vol 41, No 2 (February), 2003: pp 447-454 .
7. Cukor D et al. Depression and Anxiety in Urban Hemodialysis Patients, Clin J Am Soc Nephrol 2: 484-490, 2007
8. Cukor D, Peterson RA, Cohen SD, Kimmel PL: Depression in end-stage renal disease hemodialysis patients. *Nat Clin Pract Nephrol* 2: 678 – 687, 2006
9. Hedayati SS, Bosworth HB, Kuchibhatla M, Kimmel PL, Szczech LA: The pre- dictive value of self-reported question- naires compared to physician diagnosis of depression in end stage renal disease patients receiving chronic hemodi- aly- sis. *Kidney Int* 69: 1662–1668, 2006
10. Zachary A. Graham et al. Increasing activity levels is a promising solution to combat muscle wastage, Am J Physiol Cell Physiol 321: C40–C57, 2021.
11. Christopher W. Et al . Patients receiving maintenance dialysis have more severe functionally significant skeletal muscle wasting than patients with dialysis-inde- pendent chronic kidney disease Nephrol Dial Transplant (2006) 21: 2210–2216
12. Danielle L. Kirkman et al.The effects of intradialytic exercise on hemodialysis adequacy: A systematic review , Seminars in Dialysis. 2019;1–11
13. Paul David Stuart Brown et al. Impact of intradialytic exercise intensity on urea clearance in hemodialysis patients, Appl. Physiol. Nutr. Metab. **43**: 101–104 (2018)
14. Anubha Devagourou et al. An Experimental Study to Evaluate the Effect of Low-Intensity Intradialytic Exercises on Serum Urea, Creatinine, and Fatigue of

Chronic Kidney Disease Patients Undergoing Hemodialysis, Saudi J Kidney Dis Transpl 2021;32(5):1253-1259

15. Chia-Huei Lin et al. Effects of Intradialytic Exercise on Dialytic Parameters, Health-Related Quality of Life, and Depression Status in Hemodialysis Patients: A Randomized Controlled Trial, Int. J. Environ. Res. Public Health 2021, 18, 9205.
16. Sharlene A. Greenwood et al. Randomized Trial—PrEscription of intraDialytic exercise to improve quAlity of Life in Patients Receiving Hemodialysis, Kidney International Reports (2021) 6, 2159–2170
17. Sterky E. et al. Elderly patients on haemodialysis have 50% less functional capacity than gender- and age-matched healthy subjects.Scand J Urol Nephrol 2005;39(5):423-30.
18. Pedro Martins et al. Association between physical activity and mortality in end-stage kidney disease: a systematic review of observational studies. BMC Nephrology (2021) 22:227
19. Bündchen et al. Intradialytic exercise in end-stage renal disease: An umbrella review of systematic reviews and/or meta- analytical studies. Clinical Rehabilitation (2021) 1–17
20. Frih B et . The Effect of Interdialytic Combined Resistance and Aerobic Exercise Training on Health Related Outcomes in Chronic Hemodialysis Patients: The Tunisian Randomized Controlled StudyFront Physiol. 2017; 8: 288.
21. H.Marzougui et al. Effects of intradialytic exercise in combination with melatonin supplementation on functional capacity, postural balance, and quality of life in hemodialysis patients .Therapeutic apheresis and dialysis (2022)
22. Storer et al. Endurance exercise training during haemodialysis improves strength, power, fatigability and physical performance in maintenance haemodialysis patients.Nephrol Dial Transplant. 2005; 20: 1429-1437
23. Diesel W. et al.Isokinetic muscle strength predicts maximum exercise tolerance in renal patients on chronic hemodialysis.Am J Kidney Dis. 1990; **16**: 109-114
24. Mathew J. Clarkson et al. Exercise interventions for improving objective physical function in patients with end-stage kidney disease on dialysis: a systematic review and meta- analysis. Am J Physiol Renal Physiol 316: F856–F872, 2019
25. Trisha L. Parsons et al. Exercise Training During Hemodialysis Improves Dialysis Efficacy and Physical Performance Arch Phys Med Rehabil Vol 87, May 2006
26. Jiang Pu et al. Efficacy and safety of intradialytic exercise in haemodialysis patients: a systematic review and meta-analysis. BMJ Open 2019;9:e020633.
27. Daugirdas. J . Simplified Equations for Monitoring Kt/V, PCRn, eKt/V, and ePCRn Advances in Renal replacement Therapy Volume 2, Issue 4, May 1995, Pages 295-304
